# Identification of sources resistant to a virulent Fusarium wilt strain (VCG 0124) infecting Cavendish bananas

**DOI:** 10.1038/s41598-021-82666-7

**Published:** 2021-02-04

**Authors:** R. Thangavelu, M. S. Saraswathi, S. Uma, M. Loganathan, S. Backiyarani, P. Durai, E. Edwin Raj, N. Marimuthu, G. Kannan, R. Swennen

**Affiliations:** 1 ICAR-National Research Centre for Banana, Tiruchirappalli, 620102 India; 2 Laboratory of Tropical Crop Improvement, KU Leuven, Leuven, Belgium; 3 International Institute of Tropical Agriculture, Arusha, Tanzania

**Keywords:** Microbiology, Plant sciences

## Abstract

Bananas are vital for food security in many countries, and half of banana production relies solely on ‘Cavendish’ (AAA), which is presently threatened by the fungal pathogen *Fusarium oxysporum* f. sp. *cubense* (*Foc*) tropical race 4. This particular virulent *Foc* strain was also found to attack other banana varieties of commercial importance. As there is no single effective management practice available so far, this study was undertaken to determine resistant sources from the genotype collection available at the ICAR-National Research Centre for Banana, Tiruchirappalli, Tamil Nadu, India for direct use by farmers and/or in breeding programmes to develop resistant hybrids. A total of 258 genotypes of different ploidies and genomic constitutions were tested against *Foc* race 1 (VCG 0124). In total, 19 genotypes (AA Unique-6, BB type-2, AAA Unique-1, AAA Cavendish-1, AAB Mysore-3, AAB Pome-1, AAB Plantain-4 and AAAB-1) were found to be immune; eight genotypes (AA Unique-1, BB type-3, AAA Cavendish-1, AAB Mysore-1, AAB Unique-1, AAB Plantain-1) were highly resistant; and nine genotypes (AA Unique-1, AAA Cavendish-3, AAB Silk-1, AAB Pome-4) were resistant. The genotypes that are resistant to the virulent *Foc* race 1 (VCG 0124) strain can be exploited directly for commercialization and/or in breeding programs to develop resistant hybrids.

## Introduction

Globally, banana is one of the most important fruits and the eighth-most important food, with ~ 148 million tons produced from 135 countries. It provides staple food for > 400 million people worldwide and is vital for food security. Although there are more than 1000 varieties of banana^1^, the entire global banana industry relies solely on Cavendish varieties (subgroup AAA) with ~ 50 million tons of annual production^2^. India is the largest producer of banana, with 29.7 million tons per annum from an area of 0.802 million ha, contributing 20.1% to global production^3^. Although more than 20 varieties are commercially grown in different parts of India, Cavendish alone occupies 57% of the total area under banana cultivation^4^.

Among the production constraints, Fusarium wilt caused by the fungal pathogen *Fusarium oxysporum* f. sp. *cubense* is considered one of the most devastating diseases in banana worldwide, including India^5^. The pathogen causes yellowing of leaves, pseudostem splitting, internal vascular discolouration and eventually death of the plants^6^. It survives in the soil for decades and has wide genetic diversity within three physiological races infecting banana^4^. *Foc* race 1 attacks Gros Michel (AAA), Silk (AAB), Pisang Awak (ABB), etc., race 2 attacks ABB cooking bananas and race 4 attacks Cavendish, in addition to the varieties susceptible to race 1 and race 2. In India, 12 different vegetative compatibility groups (VCGs), 0124, 0125, 0124/0125, 0128, 0129, 01211, 01212, 01214, 01217, 01218, 01220, 01221 and 01213/01216, have been recorded in different banana varieties, and VCG 0124 of race 1 was found to attack the Cavendish banana varieties in the Theni district of Tamil Nadu^7,8^. This virulent strain has now spread to the entire banana-growing belt of Theni district, Tamil Nadu, resulting in field abandonment or the cultivation of less remunerative crops. In addition, the strain has spread to other major banana growing states of West Bengal, Madhya Pradesh, Gujarat and Maharashtra (Thangavelu, unpublished). *Foc* race 1 could also cause serious damage to the banana industry of other countries in the near future if it crosses the border.

Different management practices exist against *Foc* pathogens, but complete protection is possible only through cultivating resistant cultivars^4,6^. Hence, there is the need to identify resistant banana genotypes either for direct cultivation or for improving commercial varieties through breeding. A cross between the *Foc* race 1-susceptible genotypes ‘Sukali Ndizi’ and ‘TMB2 × 8075–7′ resulted in complete resistance against *Foc* race 1 (VCG 0124), with the single recessive gene responsible for resistance named *Panama disease* 1^9^. The present study aimed to identify sources of resistance to *Foc* race 1 (VCG 0124) to use in breeding for developing resistant varieties.

## Results

**Figure 1:**
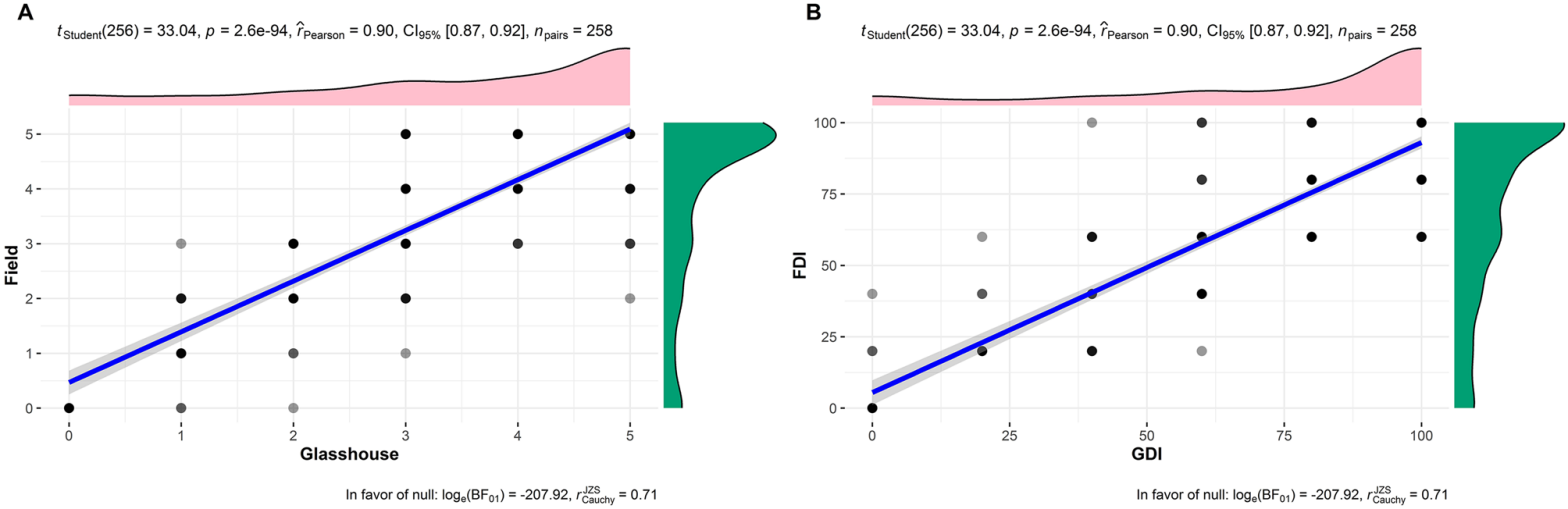
Pearson correlation analysis of the internal vascular discolouration score (**A**) and disease index (**B**) of the banana genotypes under glasshouse and field conditions.

A total of 258 genotypes belonging to different genomic groups of bananas were included in the disease screening under glasshouse and field conditions against *Foc* race 1 to accomplish the formulated objectives. Before assigning the disease reaction categories of the tested banana genotypes, the distribution of the IWDS (Internal Wilt Disease Score) was assigned based on the percentage of discoloured area in the corm on a 0–5 scale: where 0 = corm completely clean, no vascular discolouration; 1 = 1 to 5%, 2 = 6–25%, 3 = 26–50%, 4 = 51–75%, and 5 = over 75% discoloured corm)^22^ was analysed for its normality and variance in addition to mean comparisons of genotypes within a genomic group under glasshouse and field conditions by ANOVA and t-test. The IWDSs of all genotypes evaluated are presented in terms of minimum, maximum, mean and mode values with DMRT (Duncan's multiple range test) and pairwise mean comparison t-test in Supplementary Table 1 and Supplementary Figs. 1 & 2. Based on the pairwise mean comparison t-test, irrespective of genomic groups, a total of 98 genotypes showed a significant shift in resistance level (*P* > *0.05*, Supplementary Table 1), and 160 (62%) genotypes did not show a shift in resistance level between glasshouse and field conditions. The distribution of IWDSs in pooled data showed that 22.5% of genotypes (36) were classified as resistant, with maximum scores ranging from 0 to 2. Among them, 52.8% (19 genotypes) were immune to the disease, showing a maximum score of 0, 22.2% (8 genotypes) were highly resistant, with a maximum score reaching 1, and 25% (9 genotypes) had a maximum score of 2 and were classified as resistant. The remaining genotypes (77.5%; 124) were found to be susceptible, reaching a maximum score between 3 and 5. Among them, 80.6% of genotypes (124) were highly susceptible to disease, showing a maximum disease score of 5, 8.9% (11 genotypes) had a maximum score of 4 and were classified as susceptible, and 10.5% (13 genotypes) were moderately resistant, with a maximum score reaching 3. Highly significant variations were observed in the IWDSs among the genotypes (*F* = *169.9; df* = *257; P* > *0.001*) and genomic groups (*F* = *637.9; df* = *6; P* > *0.001*) tested. The overall analysis of the IWDSs showed that the AA genomic group registered disease scores ranging from 0 to 3, while AAA, AAB, AB, ABB, BB and Tetraploid (AAAB &ABBB) groups registered IWDSs of 2 to 3, 2 to 5, 4 to 5, 5, 2 to 4, and 2 to 5, respectively, under glasshouse conditions (Supplementary Fig. 2). Except for the AAA (*t* = *0.460, P* > *0.0001*), AB (*t* = *0.424, P* > *0.001*) and BB (*t* = *-0.386, P* > *0.01*) genomic groups, all other genomic groups exhibited similar disease reactions (*P* < *0.05*) under field conditions. To establish the relationship between glasshouse and field experiments, the pooled data were subjected to Pearson rank correlation analysis. The correlation coefficient of the glasshouse IWDSs (*r* = *0.90, df* = *256, P* < *0.001*) and DI (*r* = *0.90, df* = *256, P* < *0.001*) showed a highly significant correlation (Fig. 1) with the field IWDSs and DI.

**Table 1:** Number of genotypes showing different reactions to *Foc* race 1 (VCG 0124) under glasshouse and field conditions.

Genomic groups	Reaction of germplasm lines to *Foc* race 1													
	Glasshouse							Field						
	I	HR	R	MR	S	HS	Total	I	HR	R	MR	S	HS	Total
AA Unique	6	3	2	4	2	0	17	8	1	2	2	2	2	17
AAA Cavendish	1	1	7	5	0	0	14	1	2	5	4	2	0	14
AAA Unique	1	0	2	2	1	1	7	1	0	0	2	0	4	7
AAB Mysore	3	5	2	3	2	0	15	3	1	5	4	1	1	15
AAB Silk	0	0	1	3	8	10	22	0	0	1	0	1	20	22
AAB Pome	1	1	7	15	9	4	37	1	0	5	12	15	4	37
AAB Unique	0	1	0	2	0	9	12	0	1	1	3	4	3	12
AAB Plantain	4	1	0	0	0	0	5	4	1	0	0	0	0	5
AB Kunnan	0	0	0	2	6	6	14	0	0	0	0	2	12	14
AB Ney Poovan	0	0	0	0	5	4	9	0	0	0	0	0	9	9
ABB Pisang Awak	0	0	0	0	4	25	29	0	0	0	0	1	28	29
ABB Monthan	0	0	0	0	7	37	44	0	0	0	0	1	43	44
ABB Unique	0	1	0	1	0	2	4	0	0	1	0	1	2	4
BB types	2	3	3	5	5	4	22	3	5	1	3	3	7	22
Tetraploid	1	1	0	1	1	3	7	1	0	1	0	1	4	7
Total	19	17	24	43	50	105	258	22	11	22	30	34	139	258

I-Immune; HR-Highly resistant; R-Resistant; MR-Moderately resistant; S-Susceptible; and HS-Highly susceptible.

There was a highly significant variation in IWDS among the AA genotypes (17) under both glasshouse (*F* = *69.08; df* = *16; P* > *0.001*) and field (*F* = *221; df* = *16; P* > *0.001*) conditions. In the case of AB genotypes (23), a highly significant difference was registered under glasshouse (*F* = *6.457; df* = *22; P* > *0.001*) and field (*F* = *18.25; df* = *22; P* > *0.001*) conditions. Similarly, the BB genotypes also showed highly significant variation under glasshouse (*F* = *5.078; df* = *6; P* > *0.001*) and field (*F* = *18.25; df* = *6; P* > *0.001*) conditions. Of the 17 unique AA genotypes evaluated, six genotypes, viz*.*, Pisang Berlin, Tongat, Kanai Bansi, and three wild genotypes, Balukpong, Assam wild and Arunachal Pradesh wild (grouped as *Musa flaviflora*), were immune to Fusarium wilt race 1 under both glasshouse and field conditions, while cvs. Rose and Matti were highly resistant in the glasshouse but immune under field conditions. The diploids *M. acuminata* ssp. *burmannicoides* and Pisang Jari Buaya were highly resistant and resistant, respectively, under both screening conditions (Tables 1, 2 and 4). In the AB group, none of the AB Kunnan subgroup (14) or Ney Poovan subgroup (9) members were found to be resistant under glasshouse or field conditions (Tables 1 and 4). Among the 22 BB types, Manohar and Sasrabale were immune, while Bhimkol, Jungle Kela II and H-201 were highly resistant under both screening conditions. Interestingly, two types, *M. balbisiana* (508) and *M*. *balbisiana* A & N (1353), which were resistant under glasshouse conditions, exhibited immune and highly resistant reactions, respectively, under field conditions (Tables 1, 2 and 4).

**Table 2:** Diploid genotypes resistant to *Foc* race 1 (VCG 0124) under glasshouse and field conditions.

Genome	Reaction of germplasm lines to *Foc* race 1					
	Glasshouse			Field		
	I	HR	R	I	HR	R
AA Unique	Pisang Berlin (657), Tongat (380), Kanaibansi (64), Balukpong wild (1717), *M. acuminata* Assam wild (1715) *M. acuminata* Arunachal Pradesh (1731)	Cv. Rose (638), Matti (182), *M. acuminata* ssp*. burmannicoides* (642)	Pisang Jari Buaya (640), Sanna Chenkadali (201)	Cv. Rose (638), Pisang Berlin (657), Matti (182), Tongat (380), Kanai bansi (64), Balukpong wild (1717), *M. acuminata* Assam wild (1715), *M. acuminata* Arunachal Pradesh (1731)	*M. acuminata.* ssp. *burmannicoides* (642)	Hatidat (21), Pisang Jari Buaya (640)
AB Ney Poovan	0	0	0	0	0	0
AB Kunnan	0	0	0	0	0	0
BB types	Manohar (47), Sasrabale (449)	Bhimkol (597), Jungle Kela II (1913), H-201 (506)	*M. balbisiana* (508), *M. balbisiana* (A&N) (1353), Srisailam Collection (2064)	Manohar (47), Sasrabale (449), *M. balbisiana* (508)	Bhimkol (597), *M. balbisiana* (A&N) (1353), Pagalapahad wild II (1184), Jungle Kela II (1913), H-201 (506)	Jungle Kela I (1912)

I-Immune; HR-Highly resistant; R-Resistant.

**Figure 2:**
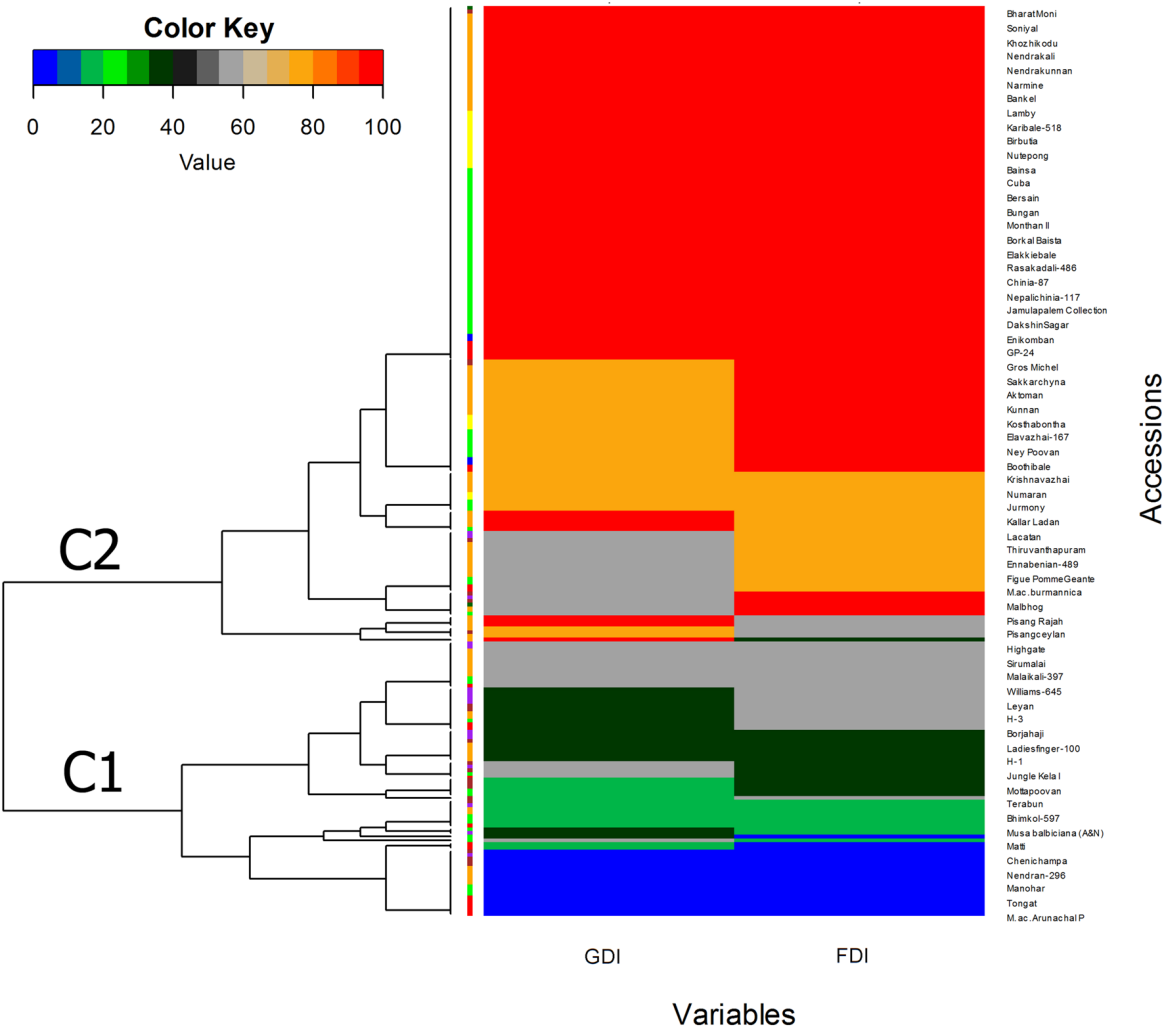
Hierarchical clustering heatmap analysis of the disease index of banana measured under glasshouse (GDI) and field (FDI) conditions against *Foc* R1.

In the case of the Cavendish genomic group (AAA) of bananas evaluated, statistically significant variation was observed among the 14 genotypes under glasshouse (*F* = *20.47, df* = *13, P* > *0.001*) and field (*F* = *44.4, df* = *13, P* > *0.001*) conditions. Among these Cavendish genotypes, Shrimanti and Manjahaji recorded immune and highly resistant reactions, respectively, under both glasshouse and field conditions, whereas Singapur was registered as resistant in glasshouse and highly resistant in field conditions (Tables 3, 4 and 5). Three lines, Williams-608, Borjahaji and GCTCV-119, exhibited resistance under both screening conditions, while Pedda Pacha, Williams and Jahaji were resistant under glasshouse conditions but moderately resistant under field conditions (Table 5 and Fig. 2). Similar to AAA Cavendish, seven unique AAA genotypes also registered significant variation under glasshouse (*F* = *67.5, df* = *6, P* > *0.001*) and field (*F* = *348.5, df* = *6, P* > *0.001*) conditions. Among the unique AAA genotypes, Red banana showed a consistent immune reaction in both screenings, while the two genotypes Kaveri Sugantham and Leyan recorded resistance under glasshouse conditions but moderate resistance under field conditions (Tables 3, 4, 5 and 2).

**Table 3:** Triploid and tetraploid genotypes resistant to *Foc* race 1 (VCG 0124) under glasshouse and field conditions.

Genome	Reaction of genotypes to *Foc* race 1					
	Glasshouse			Field		
	I	HR	R	I	HR	R
AAA Cavendish	Shrimanti (618)	Manjahaji (17)	Singapur (111), Williams (608), Borjahaji (9), GCTCV-119 (632), PeddaPacha (500), Williams (645), Jahaji (12)	Shrimanti (618)	Manjahaji (17), Singapur (111)	Williams (608), Borjahaji (9), GCTCV-119 (632), Highgate (498), Madhukar (1653)
AAA Unique	Red Banana	0	Kaveri Sugantham (1419), Leyan (71)	Red Banana	0	0
AAB Mysore	Cheeni Champa (15), Karpura Chakkarakeli (215), Kottavazhai (256)	Terabun (362), Poovan (197), Soneri (312), Mottapoovan (501), Mysorebale (619)	Borchampa (45), Alpon (78)	Cheeni Champa (15), Karpura Chakkarakeli (215), Kottavazhai (256)	Terabun (362)	Palayankodan (192), Poovan (197), Poovan (294), Soneri (312), Mottapoovan (501)
AAB Silk	0	0	Sabri (1005)	0	0	Sabri (1005)
AAB Pome	Atrusingan (497)	H-1(209)	Ladies Finger (1000), Padathi (191), Ladan Small (240), Pacha (2490), Ladan Pointed (2410), Co-1 (554), H-3 (735)	Atrusingan (497)	0	Ladies Finger (100), Padathi (191), Ladan Small (240), Pacha (249), H-1 (209)
AAB Unique	0	Popoulu (2280),	0	0	Popoulu (2280)	Kalibow ( 211)
AAB Plantain	Chengalikodan (701), Nendran (296), Nendran (615), Nedu Nendran (702)	Nijokome (731)	0	Chengalikodan (701), Nendran (296), Nendren (615), Nedu Nendran (702)	Nijokome (731)	0
ABB Pisang Awak	0	0	0	0	0	0
ABB Monthan	0	0	0	0	0	0
ABB Unique	0	Karthobiumtham (50)	0	0	0	Karthobiumtham (50)
Tetraploid (AAAB)	TMB × 5295–1 (2349)	FHIA-01 (2347)	0	TMB × 5295–1 (2349)	0	FHIA-01 (2347)
Tetraploid (ABBB)	0	0	0	0	0	0

I-Immune; HR-Highly resistant; R-Resistant.

**Table 4:** Genotypes showing resistant reactions (immune, highly resistant and resistant reactions) to *Foc* race 1 (VCG 0124) common to both glasshouse and field conditions.

Genome	Reactions of genotypes to *Foc* race 1		
	I	HR	R
AA Unique	Pisang Berlin (657), Tongat (380), Kanaibansi (64), Balukpong wild (1717), *M. acuminata* Assam wild (1715), *M. acuminata* Arunachal Pradesh (1731)	*M. acuminata* ssp. *burmannicoides* (642)	Pisang Jari Buaya (640)
AB Ney Poovan	0	0	0
AB Kunnan	0	0	0
BB types	Manohar (47), Sasrabale (449)	Bhimkol (597), Jungle Kela II (1913), H-201 (506)	0
AAA Cavendish	Shrimanti (618)	Manjahaji (17)	Williams (608), Borjahaji (9), GCTCV-119 (632)
AAA Unique	Red Banana	0	0
AAB Mysore	Cheeni Champa (15), Karpura Chakkrakeli (215), Kottavazhai (256)	Terabun (362)	0
AAB Silk	0	0	Sabri (1005)
AAB Pome	Atrusingan (497)	0	Ladies Finger (100), Padathi (191), Ladan Small (240), Pacha (249)
AAB Unique	0	Popoulu (2280)	0
AAB Plantain	Chengalikodan (701), Nendran (296), Nendran (615), Nedu Nendran (702)	Nijokome (731)	0
ABB Pisang Awak	0	0	0
ABB Monthan	0	0	0
ABB Unique	0	0	0
AAAB	TMB × 5295–1 (2349)	0	0
ABBB	0	0	0

I-Immune; HR-Highly resistant; R-Resistant.

**Table 5:** Genotypes showing shift in reaction for *Foc* race 1 (VCG 0124) from glasshouse to field conditions.

Genome	Shift in reaction		Genome	Shift in reaction	
	G*	F**		G*	F**
**AA Unique (8)**			**AAB Silk (11)**		
Cv. Rose (638), Matti (182)	HR	I	Bangladesh Malbhog (2101)	MR	S
Sanna Chenkadali (201)	R	MR	Malbhog (1), Baidi Chinia (445)	MR	HS
Hatidat (21)	MR	R	Saapkal (8), Digjowa (14), Honda (57), Amrithapani (212), Sakkarchayna (355), Ayirankarasthali (447), Pisangrajabulu (462), Madhuranga (491)	S	HS
Pisang Lilin (195), *M. acuminata* ssp. *burmannicoides* (1631)	MR	S	**AAB Pome (11)**		
Namarai (185), Anaikomban (208)	S	HS	H-1 (209)	HR	R
**AB NeyPoovan (5)**			Ladan pointed 241, H-3 (735), Co-1 (554)	R	MR
Gragricsarpara (364), Safed Velchi (458), Rasakadali (486), Rasakadali (717), Ney Poovan (623)	S	HS	Hoobale (519), Pachaladan (190), Ladan (242), Ennabenian (489), Ladies Finger (280), Padathi (537), Figue Pomme Geante(692)	MR	S
**AB Kunnan (6)**			**AAB Unique (8)**		
KNR Mutant (737)	MR	S	Thiruvanthapuram (125)	MR	S
Aktoman (53), Adukka Kunnan (147), Nattupoovan (186), Padali Moongil (482), Kunnan (178)	S	HS	Kalibow (211)	HS	R
**BB type (8)**			Pisang Rajah (217), Dudhsagar (374)	HS	MR
*Musa balbisiana* (508)	R	I	Nendrapadathi (187)	MR	S
*M. balbisiana* (A&N) (1353), Srisailam Collection (2064)	R	HR	Jwaribale (110)	HS	MR
Elavazhai (555)	MR	HS	Kullan (177), Cherapadathi (160)	HS	S
Elavazhai (167), Beejikela (1914)	S	HS	**ABB Pisang Awak (6)**		
Pagalapahad wild II (1184)	MR	HR	Kanthali (79), Chinia (347), Karpuravalli (494), Boothibale (584)	S	HS
Jungle Kela I(1912)	MR	R	Octoman (491), Deshikadali (65)	HS	S
**AAA Cavendish (8)**			**ABB Monthan (6)**		
Singapur (111)	R	HR	Bathesa Ash (246), Peykunnan (338), Peykunnan (538), Birbutia (86), Singhalaji (137), Kothia (96)	S	HS
Peddapacha (500), Williams (645), Jahaji (12)	R	MR	**ABB Unique (2)**		
Highgate (498), Madhukar (1653)	MR	R	Karthobiumtham (50)	HR	R
Dwarf Cavendish (165), Lacatan (378)	MR	S	Ginde (427)	MR	S
**AAA Unique (5)**			**AAAB (1)**		
Kaveri Sugantham (1419), Leyan (71)	R	MR	FHIA-01 (2347)	HR	R
			**ABBB (2)**		
2390–2 (670), Thellachakarakeli (166)	MR	HS	Sawai (390)	MR	S
Gros Michel (1621)	S	HS	Foconah (471)	S	HS
**AAB Mysore (11)**					
Poovan (197), Soneri (312), Mottapoovan (501)	HR	R			
Mysorebale (619)	HR	MR			
Borchampa (45), Alpon (78)	R	MR			
Palayankodan (192), Poovan (294)	MR	R			
Garomoina (43)	MR	S			
Pisang Ceylan (646)	S	MR			
Dasaman (48)	S	HS			

*F: Field conditions (Hot spot); G: Glasshouse conditions. I-Immune; HR-Highly resistant; R-Resistant; MR-Moderately resistant; S-Susceptible; HS-Highly susceptible.

With regard to the AAB genomic group, which included the Mysore, Silk, Plantain, Pome and Unique subgroups, there was highly significant variation among the genotypes screened under glasshouse (*F* = *45.07, df* = *90, P* > *0.001*) and field (*F* = *194.8, df* = *90, P* > *0.001*) conditions. Specifically, the AAB Mysore genotypes showed highly significant variation among the 15 genotypes screened under glasshouse (*F* = *59.03, df* = *14, P* > *0.001*) and field (*F* = *166.7, df* = *14, P* > *0.001*) conditions. Among them, Cheeni Champa, Karpura Chakkarakeli and Kottavazhai were immune, and Terabun was highly resistant under both screening conditions (Tables 3 and 4). The variation within the genotypes of the AAB silk genomic group was also highly significant under glasshouse (*F* = *17.13, df* = *21, P* > *0.001*) and field (*F* = *116.8, df* = *21, P* > *0.001*) conditions. Among them, interestingly, cv. Sabri was resistant under glasshouse and field conditions. The differences between the genotypes of the AAB Pome genomic group were significant under glasshouse (*F* = *20.3, df* = *36, P* > *0.001*) and field (*F* = *59.87, df* = *36, P* > *0.001*) conditions. Out of 37 AAB Pome genotypes evaluated, the genotype Atrusingan was immune, and Ladies Finger, Padathi, Ladan Small and Pacha were resistant under both testing conditions, while H-1 was highly resistant under glasshouse conditions but resistant under field conditions. In the case of 13 AAB unique genotypes, the variation in IWDSs was highly significant under glasshouse (*F* = *20.44, df* = *11, P* > *0.001*) and field (*F* = *114, df* = *11, P* > *0.001*) conditions. Among them, only Popoulu was highly resistant under both screenings, while Kalibow exhibited a shift in resistance level between field and glasshouse conditions with resistant and highly susceptible reactions, respectively (Tables 3, 4, and 5 and Fig. 2). The AAB Plantain type also registered a significant variation under glasshouse (*F* = *1.187, df* = *4, P* > *0.001*) and field (*F* = *63.33, df* = *4, P* > *0.001*) conditions. Among the AAB plantain genotypes, Chengalikodan, Nendran-296, Nendran-615 and Nedu Nendran-702 were immune, and Nijokome was highly resistant under both screening conditions.

The differences in the IWDSs of the ABB genomic group, which included the Monthan, Pisang Awak, and Unique subgroups, were statistically significant under glasshouse (*F* = *65.57; df* = *2; P* > *0.001*) and field (*F* = *148.3; df* = *2; P* > *0.001*) conditions. The differences among the genotypes of the Monthan (*F* = *5.712; df* = *43; P* > *0.001*, *F* = *21.95; df* = *43; P* > *0.001*), Pisang Awak (*F* = *1.862; df* = *29; P* > *0.01*, *F* = *8.878; df* = *29; P* > *0.001*) and Unique (*F* = *56.21; df* = *3; P* > *0.01*, *F* = *210.2; df* = *3; P* > *0.001*) subgroups were statistically significant under both screening conditions. It was observed that none of the Pisang Awak (30), Monthan (44) and Unique (4) genotypes evaluated were immune/highly resistant or resistant to *Foc* race 1, except Karthobiumtham (ABB Unique), which was highly resistant under glasshouse conditions and resistant under field conditions (Tables 1, 3 and 4).

The IWDSs among the genotypes of the tetraploids were highly significant under glasshouse (*F* = *54.24; df* = *6; P* > *0.001*) and field (*F* = *396.5; df* = *6; P* > *0.001*) conditions. Among the tetraploids, one genotype of AAAB Unique (TMB × 5295–1) exhibited an immune reaction under both conditions, while AAAB Pome (FHIA-01) showed a highly resistant reaction under glasshouse conditions and resistance under field conditions (Tables 3 and 4).

As there was a significant shift in the IWDS and DI of banana genotypes, multivariate techniques, hierarchical clustering heatmaps (HCHs) and principal coordinate analysis (PCoA), were employed to classify and visually select the genotypes based on the colour scale of disease reaction categories (i.e., Immune—I, Highly resistant—HR, Resistant—R, Moderately resistant—MR, Susceptible—S, and Highly susceptible—HS) derived from glasshouse (GDI) and field (FDI) disease indices. Based on the HCH (Fig. 2), all the banana genotypes evaluated were separated into two major clades. Clade 1 (C1, Fig. 2), consisting of 48 genotypes, was categorized into the I (blue), HR (green) and R (dark green) genotypes, while clade 2 (C2, Fig. 2) consisted of 210 genotypes, which were categorized into the MR (grey), S (orange) and HS (red) genotypes. The shift in resistance levels of certain genotypes (38%) might be due to the difference in the DI in field and glasshouse experiments. It was observed that 3.1% of the I (3), 4.1% of the HR (4), 13.3% of the R (13), 16.3% of the MR (16), 22.4% of the S (22) and 40.8% of the HS (40) genotypes categorized based on field conditions showed different disease reaction categories under glasshouse conditions.

**Figure 3:**
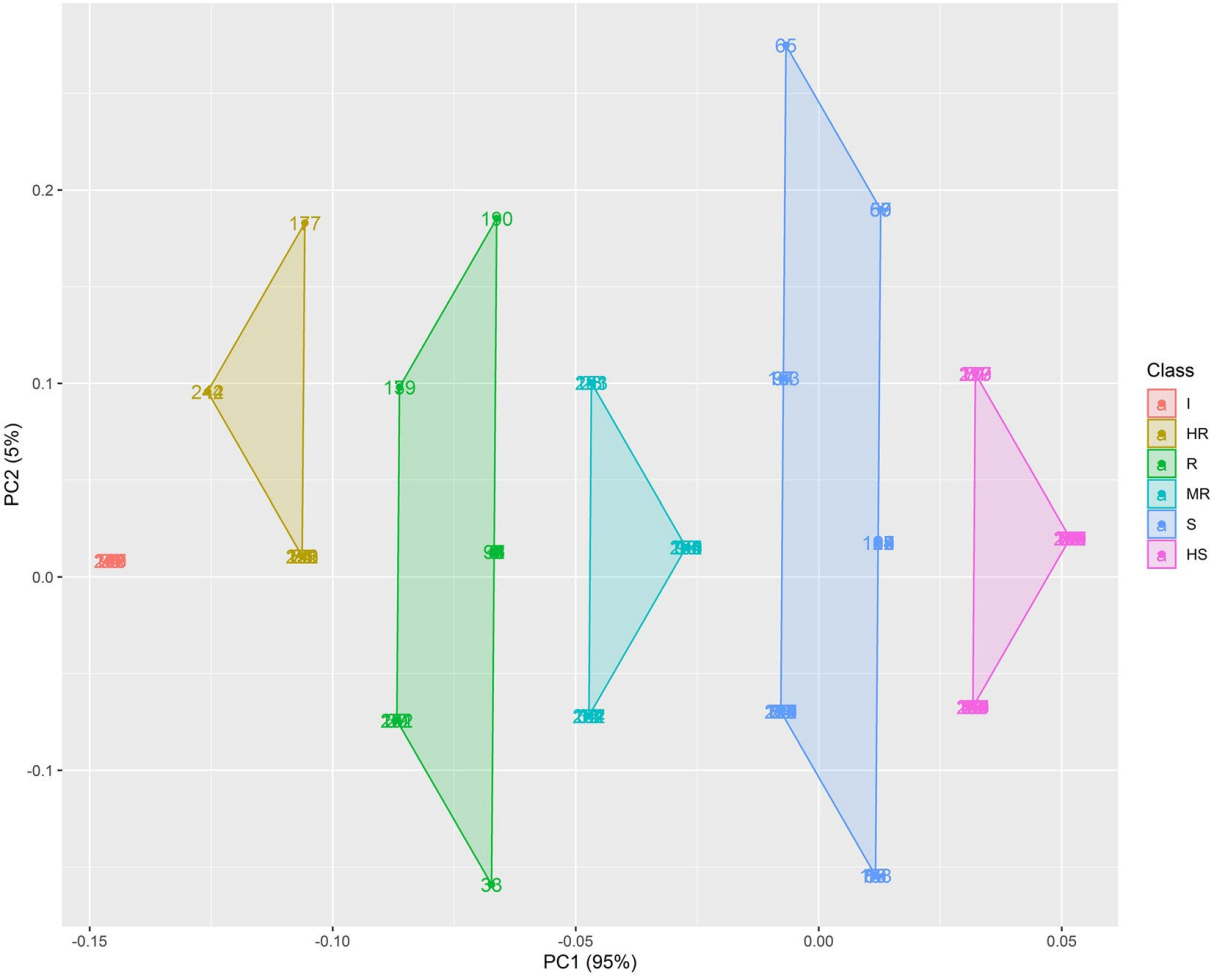
Principal coordinate analysis of the 258 banana genotypes based on the internal vascular discolouration score and disease index.

To further confirm these results, PCoA was conducted to visually determine the disease reaction categories of the 258 banana genotypes. The data of individual genotypes were shifted to be centred at zero and then normalized to obtain a unitary variance. Eigenvalues were examined to determine the extent of variation explained by the principal coordinates where FDI was principal coordinate-1 (PC1) and GDI was principal coordinate-2 (PC2). PCoA showed that PC1 accounted for 95% of the variation and that PC2 accounted for 5% of the variation. Figure 3 clearly shows different clusters that separate banana genotypes into I, HR, R, MR, S, and HS categories based on PC1 and PC2. Individual genotypes with I, HR, R and MR reactions are shown in the left part of the graph, with loads from -0.07 to -0.15 for PC1 and from -0.01 to 0.18 for PC2. Individual genotypes with S and HS reactions are in the centre to the right side of the graph, with loads from -0.01 to 0.05 for PC1 and from -0.01 to 0.28 for PC2. Red points (labelled with I) correspond to genotypes with an immune reaction (19), brown points (labelled with HR) correspond to genotypes with a highly resistant reaction (11), green points (labelled with R) correspond to genotypes with a resistant reaction (18), copper blue points (labelled with MR) correspond to genotypes with a moderately resistant reaction (30), sky blue points (labelled with S) correspond to genotypes with a susceptible reaction (42) and pink points correspond to genotypes with a highly susceptible reaction (138). Therefore, the PCoA further strengthened the results observed in the dendrogram in determining the disease reaction categories of the banana genotypes tested.

## Discussion

Among the production constraints, Fusarium wilt caused by *Foc* is a serious problem for banana worldwide^10^. Epidemics of Fusarium wilt due to *Foc* race 1 caused severe damage to Gros Michel (AAA) plantations in the twentieth century, after which the *Foc* race 1*-*resistant alternative Cavendish banana rescued the global banana industry^11,12^. Grand Naine, a Cavendish variety, contributes to 47% of global production, with an export of 22.3 million tons worth US$ 13.8 billion per annum. The production of Cavendish in Asia^13^ is also seriously threatened by the virulent *Foc* TR4^14^. This lethal strain has spread to 19 different banana-growing countries^15,16^ and is threatening the livelihoods of millions of farmers and the global banana export industry. In India, the devastation of Cavendish banana due to *Foc* race 1 (VCG 0124) was first reported in the Theni district, Tamil Nadu, in 2009^7^, and a recent survey revealed its spread to other major Cavendish growing states, such as Gujarat, Maharashtra, Madhya Pradesh and West Bengal, where 0.224 million ha of Cavendish banana produces 11.27 million tons^17^. The *Foc* race 1 strain (VCG 0124) was found to be more virulent, as it affected more commercial banana cultivars and resulted in disease symptoms ten days earlier, than other *Foc* race 1 strains. The Cavendish banana grown in Nampula, Mozambique (pers. comm. Altus Viljoen, Stellenbosch University, South Africa) and in New South Wales, Australia^18^ also succumbed to *Foc* race 1 (VCG 0124) during 2013 and 2010–2011, respectively. This indicates that *Foc* race 1 along with TR4 can also seriously challenge Cavendish banana production worldwide. In addition, this particular *Foc* race 1 is already present in most banana-growing regions of the world, causing serious damage to many other banana varieties.

Among the available strategies to combat this fungal disease^19^, cultivation of resistant varieties is considered to be the most effective method^20^. Therefore, we screened banana genotypes available in India belonging to various genomes, groups and subgroups both in glasshouse and field conditions (for main and ratoon crops) to identify sources of resistance to *Foc* race 1 for utilization either directly or for incorporation into the banana improvement programme.

**Figure 4:**
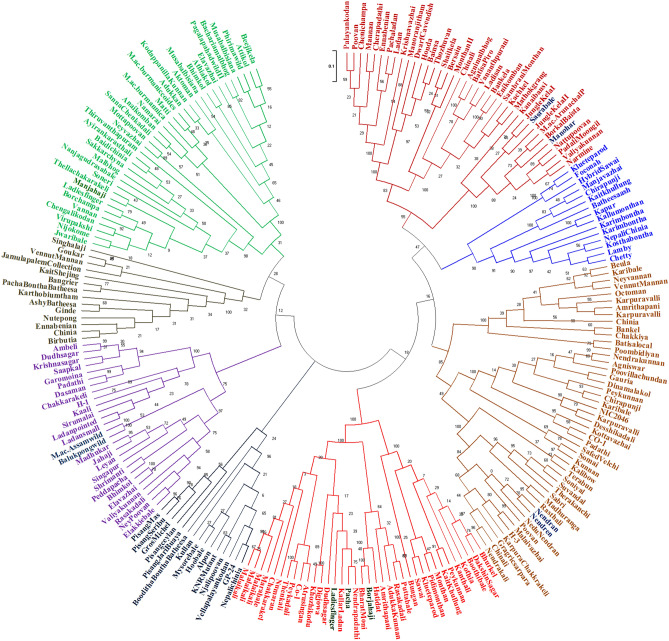
Dendrogram showing the genetic diversity and genetic relatedness among core germplasm genotypes maintained at ICAR-NRCB, Trichy, using IRAP markers. The reliability and robustness of the dendrogram were tested by bootstrap analysis using DARwin ver. 6.0.021 (https://darwin.cirad.fr/product.php) software with 1000 replications.

In the present study, 258 genotypes consisting of diploids (AA, AB, BB group and wild species), triploids (Cavendish, Plantain, Pome, Silk, Mysore, Pisang Awak, Monthan, etc.), and tetraploids (AAAB, ABBB) were evaluated, and their IWDSs and DI were statistically analysed. The results covered a wide diversity (Fig. 4). Based on the statistical analyses, 19 and 22 genotypes were immune, 17 and 11 were highly resistant and 24 and 22 were resistant to Fusarium wilt race 1 under glasshouse and field conditions, respectively. Many genotypes were consistently immune (19), highly resistant (8) and resistant (9) under both glasshouse and field conditions. They belonged to the AA Unique (8), AAA Cavendish (5), AAB Pome (5), AAB Plantain (5), AAB Mysore (4), BB type (5), AAA Unique (1), AAB Silk (1), AAB Unique (1), and AAAB groups (1). However, 98 of 258 genotypes evaluated showed a significant difference in IWDSs between glasshouse and field conditions (Supplementary Table 1). This shift in the disease reaction categories might be due to (i) the different ages of the planting material used (mature plants have an enhanced immune response and protective cellular structures such as cell wall enforcements)^21,22^; (ii) the inoculum type (the conidia used in the glasshouse are less efficient than chlamydospores generally present in the field)^23^; and (iii) the presence of multiple other biotic factors under field conditions^24,25^. Although certain genotypes showed variation between the screening conditions, the correlation analysis showed a highly positive and significant relationship based on the IWDS and DI at the 95% confidence level. This result indicates the consistency of genotype performance in addition to the possibility of the early selection method for resistance to Fusarium wilt under glasshouse conditions.

The heatmap (Fig. 2) generated in the present study based on the DI of 258 genotypes evaluated against *Foc* race 1 (VCG 0124) under both GDI and FDI conditions showed the presence of two major clades, viz., C1 and C2. In general, C1 contained immune, highly resistant and resistant genotypes, and C2 contained moderately resistant, susceptible and highly susceptible genotypes. This is in accordance with the results of the PCoA, indicating that the results observed in the dendrogram were similar to those of the PCoA in determining the disease reaction categories (Fig. 3). As both analyses clearly distinguished all the tested banana genotypes into different resistance categories, one can visually select the genotypes of interest based on the colour scale and progression of the symptom values^26^.

Although a differential (immune to susceptible) reaction was observed in certain genome groups evaluated, only a susceptible reaction was recorded in all AB genotypes (Kunnan and NeyPoovan types), all ABB genotypes (Pisang Awak, Monthan and Unique) and one tetraploid ABBB tested under both glasshouse and field conditions. Hence, one could hypothesize that genotypes with 50% or more of the B genome (B- and B-rich genotypes) might be especially susceptible to *Foc* race 1.

**Figure 5:**
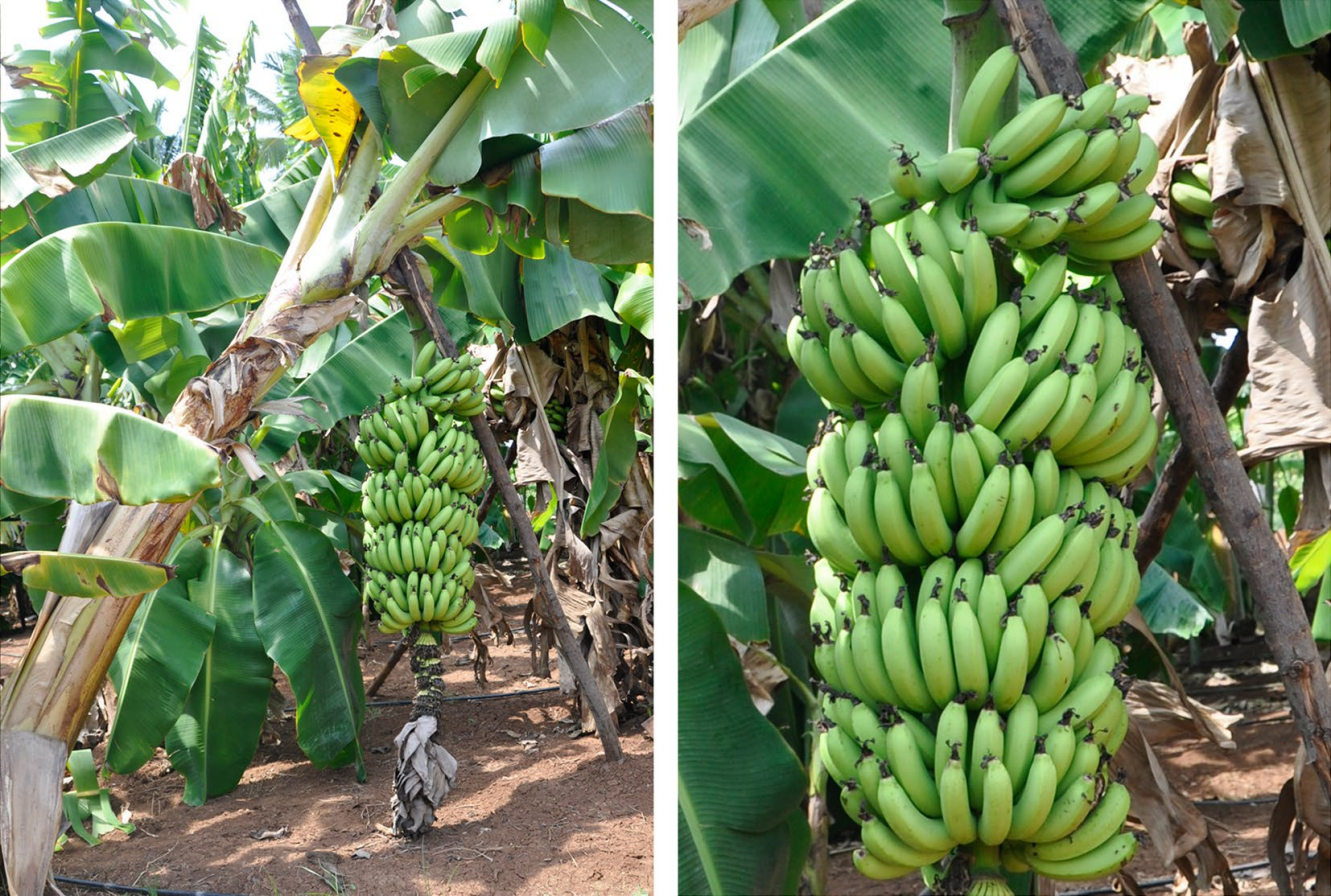
Cavendish genotype cv. Manjahaji, which was resistant to *Foc* race 1 (VCG 0214) under field conditions.

As India is extensively under Cavendish banana production, 14 Cavendish ecotypes have been selected, among which the important ecotypes are Shrimanti, Manjahaji, Singapur, Borjahaji, Jahaji, Williams, Madhukar, Pedda Pacha, etc.,^27^. Out of the 14 Cavendish genotypes evaluated, five genotypes, Shrimanti (immune), Manjahaji (highly resistant; Fig. 5), Williams, Borjahaji, and GCTCV-119 (resistant), showed differences in the degrees of resistance under both glasshouse and field conditions. Based on earlier reports, the dwarf variant of Cavendish, Dwarf Parfitt and GCTCV 119^14,28^ were resistant to *Foc* TR4, while the present study showed resistance to *Foc* race 1. The AAA Cavendish cultivars Shrimanti (immune), Manjahaji (highly resistant), Williams and Borjahaji (resistant) grouped together in one clade in the diversity studies of Cavendish types using IRAP (Fig. 4) and RAPD markers^27^. Red Banana (AAA-Red Dacca), which is being grown in different parts of the world, including India, was found to be consistently immune under both glasshouse and field conditions. Due to Cavendish succumbing to *Foc* race 1 (VCG 0124) in India, this variety is being widely cultivated to replace Grand Naine in the hot spot area of Theni district, Tamil Nadu.

Among the 23 AB diploid genotypes (Kunnan-14 and NeyPoovan-9), none were found to exhibit *Foc* resistance. Their morpho-molecular characterization^29^ suggested that they might have evolved from the same ancestors (Fig. 4), which lacked *Foc* race 1 resistance. NeyPoovan (AB) has much commercial importance and is grown extensively in all southern states of India and most of the East African countries where it is called Sukali-Ndizi. Breeding NeyPoovan is difficult due to its female sterility^30^, but Kunnan has residual fertility^31^.

Out of the 15 Mysore subgroups among the AAB genotypes, three genotypes, Cheeni Champa, Karpura Chakarakeli and Kottavazhai, were immune, and Terabun was highly resistant to *Foc* race 1 under both glasshouse and field conditions. Of these, Kottavazhai is not commercially cultivated, as it exhibits enlarged ovules resembling seeds that are not preferred by consumers. Thus, Cheeni Champa and Karpura Chakarakeli, which are generally high yielders, could replace the other Mysore types of bananas.

Among the 22 genotypes belonging to the Silk subgroups (AAB), only Sabri showed resistance to *Foc* race 1 under both glasshouse and field conditions. As Sabri is an ecotype of Rasthali, a niche cultivar of the northeastern state, Tripura, this genotype can replace the other Silk genotypes grown in the southern states of India. Among the 37 genotypes of the Pome subgroup, only Atrusingan showed an immune reaction but has no commercial value due to its poor bunch quality. Its residual female fertility offers scope for improvement through breeding. Among the AAB Pome types, Ladies Finger and Pacha were found to be resistant and clustered together in one microcluster sharing > 80% similarity based on the IRAP diversity analysis^29^.

None of the AAB Unique genotypes exhibited resistance against *Foc* race 1, except Popoulu, an introduced banana genotype. These genotypes could be used for varietal rotation. Interestingly, all five plantain types were found to be immune to highly resistant. AAB plantains such as Nendran (296 and 615), which were immune in the present study, clustered together (Fig. 4) in the IRAP-based diversity study^29^. Their morpho-molecular characterization^29^ suggested the possibility of common ancestors to plantains. The resistant plantains are being grown commercially and have replaced Grand Naine in the *Foc* race 1 endemic area of Tamil Nadu in India.

Within the ABB genotypes of the Pisang Awak, Monthan and Unique subgroups, only Karthobiumtham was highly resistant and resistant to *Foc* race 1 in the glasshouse and field conditions, respectively. As this genotype is also resistant to the root-lesion nematode *Pratylenchus coffeae*^32^, it can be used as a good source of resistance to Fusarium wilt and *P. coffeae*.

Among the 17 AA Unique genotypes, eight genotypes consistently showed either immune, highly resistant or resistant reactions to *Foc* race 1 under glasshouse and field conditions. However, the use of Pisang Berlin, Tongat and Hatidat in breeding is limited due to their poor bunch and fruit qualities. Wild AA diploids such as Balukpong and *M. acuminata*-Assam, which were immune in the present study, were grouped in the same clade in the IRAP diversity studies (Fig. 4)^29^. Among the wild genotypes (seeded) with an immune reaction to *Foc* race 1, *M. acuminata* ssp. *burmannicoides* (also called Calcutta 4) is extensively used in breeding programmes worldwide^33,34^. Pisang Jari Buaya with multiple resistances is also used extensively across various banana breeding programs, resulting in several promising hybrids^35,36^. In the Indian banana breeding programme, however, it produces few seeds and progenies. Matti, an important AA parthenocarpic diploid, and a landrace in southern India showed immune and highly resistant reactions under field and glasshouse screenings, respectively. Many improved diploids have been developed using Matti as one of the parents in the breeding programme^37,38^.

In the present study, five out of 22 *M. balbisiana* genotypes showed consistently resistant reactions against *Foc* race 1. Wild BB diploids Manohar and Sasrabale were immune in the present study and clustered in an IRAP-based diversity study^29^. Among these, Bhimkol, a soft seeded type valued for its rich nutritional and therapeutic value, is grown extensively in the backyards of the hilly northeastern region of India. Considering the importance of this genotype, efforts are being made to develop a seedless Bhimkol, but progress is slow due to its long crop duration (18–24 months). Bhimkol is also less susceptible to stem weevils^39^; hence, this genotype could be used in breeding to develop improved parthenocarpic diploids with multiple resistance.

In general, this screening study of diverse banana genotypes against *Foc* race 1 infecting Cavendish and, more particularly, the evaluation of AA and BB diploid genotypes indicates that wild (*M. acuminata* and *M. flaviflora*) and AA parthenocarpic genotypes exhibit much better resistance than *M. balbisiana* types. Approximately 64% of AA diploid genotypes have exhibited resistant, highly resistant or immune reactions to *Foc* race 1, while only 36% of the *M. balbisiana* genotypes did so.

## Conclusions

Our findings demonstrate the value of evaluating the genotypes present in India against the endemic *Foc* race 1 strain for the sustainable management of Fusarium wilt disease. Both screenings in the glasshouse and fields are useful. Glasshouse screening is fast and highly correlated with field methods, where varieties are classified either at the same level of response or are slightly less/highly resistant. It is encouraging to find that among the 14 Cavendish varieties tested, five Cavendish genotypes showed potential to continue the production of Cavendish: Shrimanti (immune), Manjahaji (highly resistant), Williams, Borjahaji, and GCTCV-119 (resistant). Additionally, more sources of resistance were found in triploids, which can be used for cultivation, and diploids, which are interesting germplasms for breeding.

## Materials and methods

### Plant material

As a National Active Germplasm Site (NAGS) for Banana, the ICAR-National Research Centre for Banana, Tiruchirappalli, Tamil Nadu, India conserves 374 genotypes in both in vitro and in vivo gene banks. Natural introgression of *Musa acuminata* (AA) with hardy wild *M. balbisiana* (BB) resulted in the development of a broad spectrum of genomic groups ranging from diploids to tetraploids (AA, AB, BB, AAA, AAB, ABB, AABB, ABBB, etc.). Of these, 258 genotypes (Supplementary Table 1) were included in this study. They consisted of different genomic groups and subgroups, such as AA Unique (genotypes that do not fit into any of the already existing subgroups in each genomic group of the Musalogue are referred to as ‘Unique’) (17), AB Kunnan (14), AB Ney Poovan (9), BB type (22), AAA Cavendish (14 which include 4 dwarf genotypes, 3 medium genotypes, and 7 tall genotypes), AAA Unique (7), AAB Mysore (15), AAB Silk (22), AAB Pome (37), AAB Unique (12), AAB Plantain (5), ABB Pisang Awak (29), ABB Monthan (44), ABB Unique (4) and AAAB/ABBB tetraploids (6) (Table 1 and Fig. 4).

### Pathogen isolation

The Fusarium wilt pathogen *Fusarium oxysporum* f. sp. *cubense* race 1 (VCG 0124) was isolated from dried vascular strands of wilt-infected Cavendish banana (cv. Grand Naine-AAA) using 25% strength potato dextrose agar (PDA) medium^7^. The single spore culture obtained was maintained on carnation leaf agar medium^40^ for immediate use, and for long-term use, the culture was stored on dried filter papers at 4°C^41^. The pathogenicity of the fungus was tested by inoculating *Foc* race 1 into potted tissue-cultured Grand Naine plants under glasshouse conditions. The pathogen was multiplied in sand maize (19:1) medium and used for inoculation of plants in the glasshouse.

### Glasshouse evaluation

Disease-free suckers weighing approximately 1.5 to 2 kg were extracted from healthy mother plants of all banana genotypes and subjected to paring and pralinage (dipping the pared suckers in Triazophos-40% EC at 0.5% conc. for 30 min) treatments to protect them from nematodes and insect pests. These treated suckers were planted in mud pots (30 × 30 × 15 cm) filled with a sterilized potting mixture containing red earth, sand and cattle manure in equal proportions. Suckers grew for 30 days with regular watering. Thereafter, approximately 30 g of sand maize *Foc* inoculum (*Foc* R1 VCG 0124) containing approximately 10^6^ cfu g^-1^ was applied to the soil around the plants in each pot. The experiment was conducted in a completely randomized design and with ten plants (i.e., replications) per genotype. Five months after *Foc* inoculation, the plants were pulled out, and disease evaluation was carried out based on the percentage of discoloured area in the corm on a 0–5 scale, where 0 = corm completely clean, no vascular discolouration; 1 = 1 to 5%, 2 = 6–25%, 3 = 26–50%, 4 = 51–75%, and 5 = over 75% discoloured corm^22^.

### Field evaluation

All genotypes were evaluated for two cycles in 2016–17 and 2017–18 in the Theni district of Tamil Nadu, India. The selected commercial plantation site was abandoned, as more than 90% of the Cavendish plants there exhibited Fusarium wilt. The red loamy soil of the study area had a pH of 6.2. The average annual rainfall of the area was 1,000 mm with an ambient temperature of 20.3–38.6 °C and RH of 37–80%. The suckers were prepared for glasshouse planting and were planted at a spacing of 2 × 2 m. The experiments were conducted in a completely randomized block design, with each genotype represented by ten replications for each cycle. Timely application of fertilizers, manures, water and other cultural operations were followed according to standard production practices^42^. The observation of the IWDS was taken at the end of each cycle based on the percentage of discoloured area in the corm at the time of harvest or when the plants were severely infected with wilt disease as described earlier. Based on these observations, the wilt index was calculated as follows: Disease Index (DI) = (average disease score of each plant/the highest disease index value) × 100^22^. The disease index was calculated for both the main and ratoon crops, and the average values of pooled samples are presented. Based on the DI, the genotypes were classified into six disease reaction categories as described in Zuo et al.^22^ with minor modifications. The reactions were immune (PDI: 0), highly resistant (PDI: > 0–20), resistant (PDI: > 21–40), moderately resistant (PDI: > 41–60), susceptible (PDI: > 61–80) and highly susceptible (PDI: > 81–100).

### Genetic diversity of banana genotypes using IRAP marker

The genomic DNA of the 258 banana genotypes was extracted from cigar leaf samples following the CTAB method^43^ and inter-retrotransposon amplified polymorphism (IRAP) analysis was performed using long terminal repeat (LTR) primers for banana as reported by Saraswathi et al.^29^. The PCR-amplified products were scored as the presence (1) or absence (0) of bands in a binary format and analysed using NTYSYS 2.01i. A genetic similarity (GS) matrix was then used in hierarchical cluster analysis using the unweighted pair group method with arithmetic averages (UPGMA) and sequential agglomerative hierarchical and nested (SAHN) clustering methods (NTSYS statistical package, Rohlf, 1990) to produce a dendrogram. The reliability and robustness of the dendrogram were tested by bootstrap analysis using DARwin software ver. 6.0.021 (https://darwin.cirad.fr/product.php) with 1000 replications.

### Statistical analysis

All analyses and plot generation took place in R, version 3.6.1, using the “Rbase 3.6.1”^44^ “dplyr 1.8.6”^45^ and “ggplot2 3.3.2”^46^ packages. To describe the difference in the IWDSs of the banana genotypes against *Foc* race 1, the distribution of the IWDSs (0–5 scale) under both glasshouse and field experiments was analysed separately. To confirm the assumption of normality in the disease scale within a genotype, the Shapiro–Wilk test was used with untransformed data using the R function “shapiro.test()”. The homogeneity of the variance across experiments and genomic groups was assessed using Bartlett's test, as implemented in the R function “var.test()”. The mean IWDS of a genotype was assumed if a genotype had a significantly (*P* > *0.05*) higher frequency of occurrence analysed by χ^2^ (chi square) test using the R function “chisq.test()”. In the case of field experiments, the data from the first and ratoon crops were pooled and analysed in every instance. Furthermore, the difference between the genotypes within a genome group was elucidated by comparing the mean IWDS and DI of the genotypes by one-way analysis of variance (ANOVA) using the R function “aov()”, where replications were included as a random variable to account for the nonindependence of successive counts from the same genotype followed by DMRT using the “agricolae 1.3–3”^47^ package with the “duncan.test” function. The difference in the mean IWDS of banana genotypes versus experimental conditions (i.e., glasshouse and field) was tested with two-sample paired Tukey's HSD test using the R function “Tukey HSD()”, where *P* > *0.05* is considered significant, which indicates a shift in the resistant reaction. The concurrence between the glasshouse and field in terms of IWDS and DI was established separately by the Pearson correlation coefficient (r) using the “ggstatsplot 0.6.1”^48^ package with the “grouped_gghistostats” function since the data were normally distributed (according to the Shapiro–Wilk test for normality). The multivariate techniques HCH and PCoA were used to classify and visually select the genotypes based on the colour scale of the disease reaction category in a three-dimensional space (x, y and z axes), where the DIs measured under both the glasshouse (GDI) and field (FDI) conditions were centred and scaled before performing the PCoA. The HCH was drawn using the “heatmap.2” function of the “gplots 3.6.3”^49^ package, and the PCoA was drawn using the “prcomp” followed by the “autoplot” function of the “ggfortify 0.4.11”^50^ packages based on the distance matrix of the genotypes. The colour code for the HCH of the DI was blue (immune-I), green (highly resistant-HR), dark green (resistant-R), grey (moderately resistant-MR), orange (susceptible-S), and red (highly susceptible-HS).

## Supplementary Information

Supplementary Information 3.

Supplementary Information 4.

## Data Availability

All data generated or analysed during this study are included in this published article.
