# Supplementary material for: Identification of sources resistant to a virulent Fusarium wilt strain (VCG 0124) infecting Cavendish bananas
Source: Sci Rep. 2021 Feb 4;11:3183. doi: 10.1038/s41598-021-82666-7 (PMC7862490; doi:10.1038/s41598-021-82666-7)
Supplement: Supplementary file 1 — Supplementary Information 3. [file 41598_2021_82666_MOESM1_ESM.doc]

# Identification of sources resistant to a virulent Fusarium wilt strain (VCG 0124) infecting Cavendish bananas

R. Thangavelu,^1*#^ M.S. Saraswathi,^1#^ S. Uma,^1#^ M. Loganathan,^1†^ S. Backiyarani,^1†^ P. Durai,^1†^ E. Edwin Raj^1†^, N. Marimuthu, ^1†^ G. Kannan^1†^ and R. Swennen^2, 3†^

^1^ICAR-National Research Centre for Banana, Tiruchirappalli-620102, India

^2^Laboratory of Tropical Crop Improvement, KU Leuven, Belgium

^3^International Institute of Tropical Agriculture, Arusha, Tanzania

*Corresponding Author

E-mail: rtbanana@gmail.com

^#^These authors contributed equally

^†^These authors also contributed equally to this work.


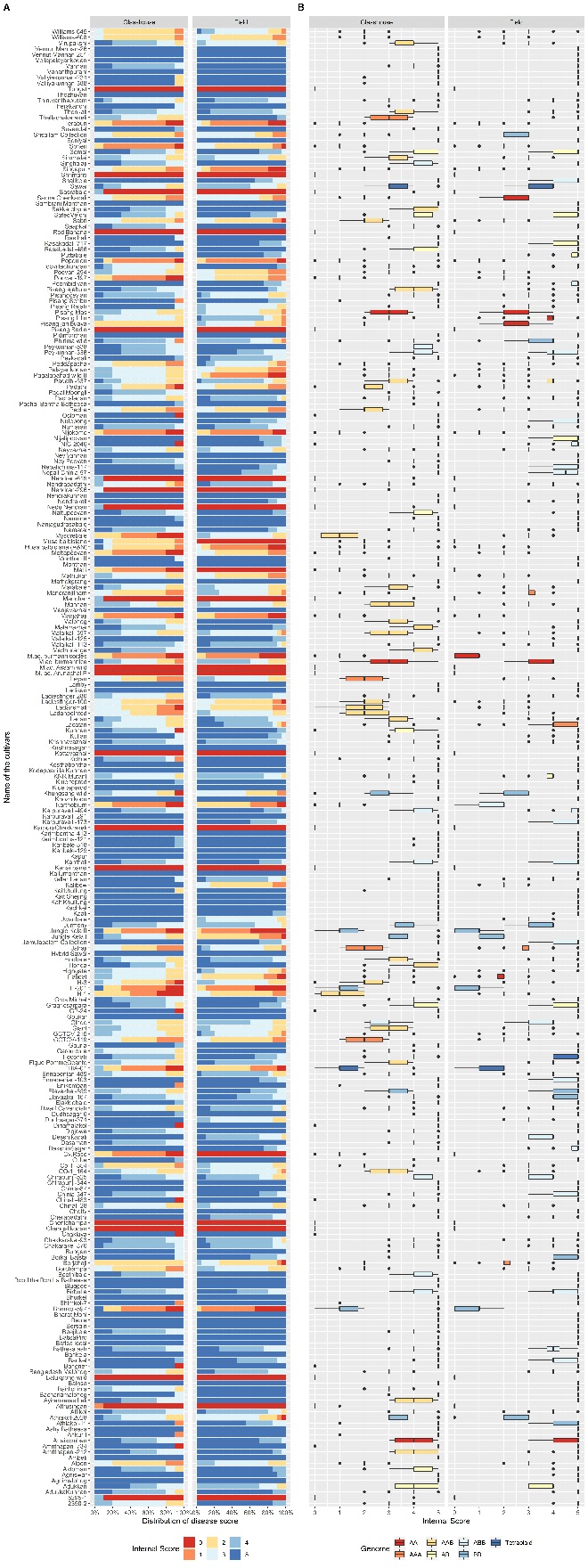


**Figure S1**: Internal Fusarium Wilt Disease score distribution and box plot of the banana genotypes. Colour in the percentage bar diagram (A) shows the distribution of disease score (0-5 scale) and box plot shows the mean (B) of the banana genotypes. Where the whiskers in the box plot represent the minimum and maximum, the box represents the 25^th^ and 75^th^ percentiles (lower and upper quartiles, respectively), the centre line represents the 50^th^ percentile and the dots represents outliers.


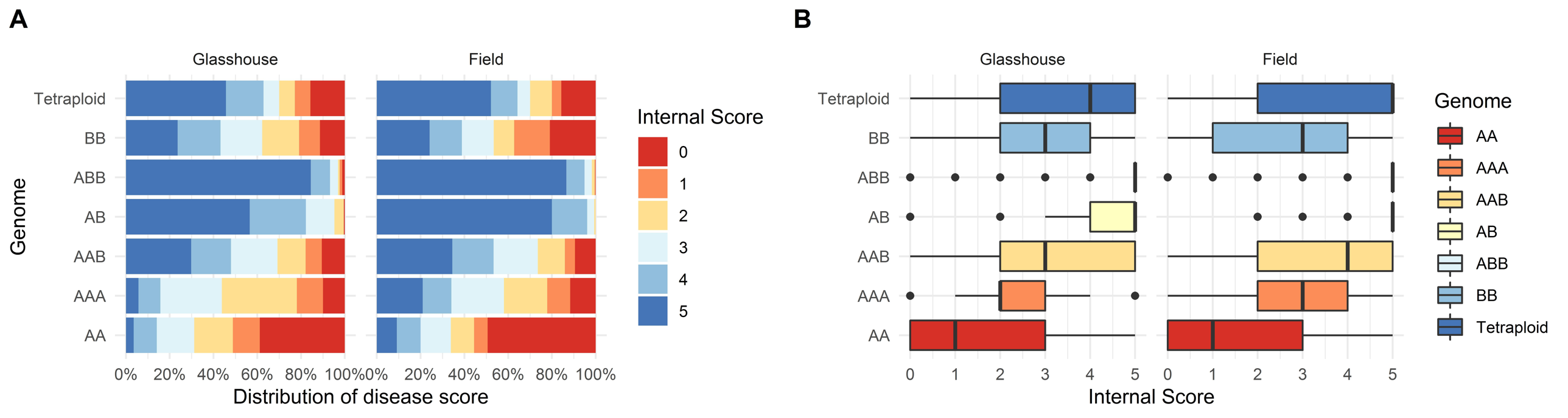


**Figure S2**: Distribution of internal Fusarium Wilt disease score according to banana genomic groups. Colour in the percentage bar diagram (A) shows the distribution of disease score (0-5 scale) and box plot shows the mean (B) of the banana genomic groups. Where the whiskers in the box plot represent the minimum and maximum, the box represents the 25^th^ and 75^th^ percentiles (lower and upper quartiles, respectively), the centre line represents the 50^th^ percentile and the dots represents outliers.
