# Supplementary material for: Identification of sources resistant to a virulent Fusarium wilt strain (VCG 0124) infecting Cavendish bananas
Source: Sci Rep. 2021 Feb 4;11:3183. doi: 10.1038/s41598-021-82666-7 (PMC7862490; doi:10.1038/s41598-021-82666-7)
Supplement: Supplementary file 2 — Supplementary Information 4. [file 41598_2021_82666_MOESM2_ESM.doc]

**Supplementary Table 1:** Disease score and disease index with disease reaction categories of different banana genotypes evaluated under glasshouse and field conditions with statistical analysis of DMRT and t-test.

| **Name** | **Glasshouse** | | | |  | **Field** | | | | |  |  | **Glasshouse** | | **Field** | |
| --- | --- | --- | --- | --- | --- | --- | --- | --- | --- | --- | --- | --- | --- | --- | --- | --- |
|  | **Min** | **Max** | **Mean** | **Mode** |  | **Min** | **Max** | **Max** | **Mean** | **Mode** | **t-test** | **Sig.** | **DI** | **DR** | **DI** | **DR** |
| **AA Unique** |  |  |  |  |  |  |  |  |  |  |  |  |  |  |  |  |
| cv. Rose | 0 | 2 | 1±0.7d | 1 |  | 0 | 0 | 0 | 0±0f | 0 | -1.00 | 0.00 | 20±7d | HR | 0±7f | I |
| Pisang Berlin | 0 | 0 | 0±0e | 0 |  | 0 | 0 | 0 | 0±0f | 0 | 0 | NA | 0±0e | I | 0±0f | I |
| Matti | 0 | 2 | 1±0.7d | 1 |  | 0 | 0 | 0 | 0±0f | 0 | -1.00 | 0.00 | 20±7d | HR | 0±7f | I |
| Tongat | 0 | 0 | 0±0e | 0 |  | 0 | 0 | 0 | 0±0f | 0 | 0 | NA | 0±0e | I | 0±0f | I |
| Kanai bansi | 0 | 0 | 0±0e | 0 |  | 0 | 0 | 0 | 0±0f | 0 | 0 | NA | 0±0e | I | 0±0f | I |
| Balukpong wild | 0 | 0 | 0±0e | 0 |  | 0 | 0 | 0 | 0±0f | 0 | 0 | NA | 0±0e | I | 0±0f | I |
| *M. ac.* Assam wild | 0 | 0 | 0±0e | 0 |  | 0 | 0 | 0 | 0±0f | 0 | 0 | NA | 0±0e | I | 0±0f | I |
| *M. ac.* Arunachal Pradesh | 0 | 0 | 0±0e | 0 |  | 0 | 0 | 0 | 0±0f | 0 | 0 | NA | 0±0e | I | 0±0f | I |
| *M. ac*. *burmannicoides* | 0 | 2 | 1±0.7d | 1 |  | 0 | 2 | 2 | 0.8±0.6e | 1 | -0.20 | 0.42 | 20±7d | HR | 20±7e | HR |
| Hatidat | 2 | 4 | 3±0.7b | 3 |  | 0 | 3 | 3 | 1.7±0.7d | 2 | -1.30 | 0.00 | 60±7b | MR | 40±7d | R |
| Pisang Jari Buaya | 2 | 2 | 2±0c | 2 |  | 1 | 4 | 4 | 2.7±0.7c | 3 | 0.70 | 0.01 | 40±0c | R | 60±0c | MR |
| Pisang Mas | 2 | 4 | 3±0.8b | 3 |  | 1 | 4 | 4 | 2.8±0.7c | 3 | -0.25 | 0.40 | 60±8b | MR | 60±8c | MR |
| Sanna Chenkadali | 1 | 3 | 2±0.7c | 2 |  | 1 | 3 | 3 | 2.6±0.8c | 3 | 0.55 | 0.06 | 40±7c | R | 60±7c | MR |
| Pisang Lilin | 2 | 4 | 3±0.5b | 3 |  | 1 | 5 | 5 | 3.6±0.9b | 4 | 0.60 | 0.07 | 60±5b | MR | 80±5b | S |
| *M. ac. burmannica* | 1 | 5 | 3±1.2b | 3 |  | 2 | 4 | 4 | 3.4±0.8b | 4 | 0.40 | 0.28 | 60±12b | MR | 80±12b | S |
| Namarai | 3 | 5 | 4±0.7a | 4 |  | 3 | 5 | 5 | 4.7±0.7a | 5 | 0.70 | 0.01 | 80±7a | S | 100±7a | HS |
| Anaikomban | 3 | 5 | 4±0.8a | 4 |  | 3 | 5 | 5 | 4.7±0.6a | 5 | 0.65 | 0.02 | 80±8a | S | 100±8a | HS |
| **AB Ney Poovan** |  |  |  |  |  |  |  |  |  |  |  |  |  |  |  |  |
| Elakkiebale | 4 | 5 | 4.9±0.3a | 5 |  | 5 | 5 | 5 | 5±0a | 5 | 0.10 | 0.16 | 100±3a | HS | 100±3a | HS |
| Nijalipoovan | 5 | 5 | 5±0a | 5 |  | 3 | 5 | 5 | 4.7±0.6b | 5 | -0.35 | 0.07 | 100±0a | HS | 100±0b | HS |
| Gragricsarpara | 2 | 5 | 4.1±1.0b | 5 |  | 3 | 5 | 5 | 4.7±0.6b | 5 | 0.55 | 0.07 | 100±10b | HS | 100±10b | HS |
| Puttabale | 5 | 5 | 5±0a | 5 |  | 3 | 5 | 5 | 4.7±0.6ab | 5 | -0.30 | 0.11 | 100±0a | HS | 100±0ab | HS |
| Safet Velchi | 2 | 5 | 4±0.9b | 4 |  | 3 | 5 | 5 | 4.5±0.7b | 5 | 0.50 | 0.11 | 80±9b | S | 100±9b | HS |
| Rasakadali-486 | 2 | 5 | 4.1±1.0b | 5 |  | 5 | 5 | 5 | 5±0a | 5 | 0.90 | 0.00 | 100±10b | HS | 100±10a | HS |
| Somai | 2 | 5 | 4.1±1.0b | 5 |  | 3 | 5 | 5 | 4.7±0.6b | 5 | 0.55 | 0.07 | 100±10b | HS | 100±10b | HS |
| Ney Poovan | 3 | 5 | 4.1±0.6b | 4 |  | 5 | 5 | 5 | 5±0a | 5 | 0.90 | 0.00 | 80±6b | S | 100±6a | HS |
| Rasakadali-717 | 5 | 5 | 5±0a | 5 |  | 3 | 5 | 5 | 4.7±0.6b | 5 | -0.35 | 0.07 | 100±0a | HS | 100±0b | HS |
| **AB Kunnan** |  |  |  |  |  |  |  |  |  |  |  |  |  |  |  |  |
| KNR Mutant | 2 | 4 | 3.1±0.6e | 3 |  | 2 | 4 | 4 | 3.7±0.6b | 4 | 0.60 | 0.01 | 60±6e | MR | 80±6b | S |
| Adukkan | 3 | 5 | 4.1±0.9bc | 5 |  | 2 | 4 | 4 | 3.6±0.7b | 4 | -0.50 | 0.10 | 100±9bc | HS | 80±9b | S |
| Aktoman | 2 | 5 | 4±0.9bc | 4 |  | 4 | 5 | 5 | 4.9±0.4a | 5 | 0.85 | 0.00 | 80±9bc | S | 100±9a | HS |
| Adukka Kunnan | 2 | 5 | 3.9±0.9cd | 4 |  | 4 | 5 | 5 | 4.9±0.4a | 5 | 0.95 | 0.00 | 80±9cd | S | 100±9a | HS |
| Nattupoovan | 3 | 5 | 4.1±0.7bc | 4 |  | 4 | 5 | 5 | 4.9±0.4a | 5 | 0.75 | 0.00 | 80±7bc | S | 100±7a | HS |
| Kodappanilla Kunnan | 5 | 5 | 5±0a | 5 |  | 5 | 5 | 5 | 5±0a | 5 | 0 | 1.00 | 100±0a | HS | 100±0a | HS |
| Padali Moongil | 3 | 5 | 4±0.7bc | 4 |  | 5 | 5 | 5 | 5±0a | 5 | 1 | 0.00 | 80±7bc | S | 100±7a | HS |
| Poovillachundan | 2 | 5 | 3.2±0.8de | 3 |  | 4 | 5 | 5 | 4.9±0.3a | 5 | 1.70 | 0.00 | 60±8de | MR | 100±8a | HS |
| Nendrakunnan | 5 | 5 | 5±0a | 5 |  | 5 | 5 | 5 | 5±0a | 5 | 0 | 1.00 | 100±0a | HS | 100±0a | HS |
| Agniswar | 5 | 5 | 5±0a | 5 |  | 4 | 5 | 5 | 4.9±0.3a | 5 | -0.10 | 0.32 | 100±0a | HS | 100±0a | HS |
| Kunnan | 0 | 5 | 3.6±1.4cde | 4 |  | 4 | 5 | 5 | 4.9±0.3a | 5 | 1.30 | 0.00 | 80±14cde | S | 100±14a | HS |
| Valiyiakunnan-234 | 2 | 5 | 4.7±0.9ab | 5 |  | 5 | 5 | 5 | 5±0a | 5 | 0.30 | 0.16 | 100±9ab | HS | 100±9a | HS |
| Valiiyakunnan-388 | 2 | 5 | 4.7±0.9ab | 5 |  | 5 | 5 | 5 | 5±0a | 5 | 0.30 | 0.16 | 100±9ab | HS | 100±9a | HS |
| Narmine | 4 | 5 | 4.9±0.3a | 5 |  | 5 | 5 | 5 | 5±0a | 5 | 0.10 | 0.16 | 100±3a | HS | 100±3a | HS |
| **BB** |  |  |  |  |  |  |  |  |  |  |  |  |  |  |  |  |
| Manohar | 0 | 4 | 0.4±1.3e | 0 |  | 0 | 0 | 0 | 0±0f | 0 | -0.40 | 0.16 | 0±13e | I | 0±0f | I |
| Sasrabale | 0 | 4 | 0.4±1.3ab | 0 |  | 0 | 0 | 0 | 0±0c | 0 | -0.40 | 0.16 | 0±13ab | I | 0±0c | I |
| *Musa balbisiana* | 1 | 5 | 2.3±1.1e | 2 |  | 0 | 0 | 0 | 0±0f | 0 | -2.30 | 0.00 | 40±11e | R | 0±11f | I |
| Bhimkol-597 | 0 | 5 | 1.4±1.4ab | 1 |  | 0 | 2 | 2 | 0.7±0.6c | 1 | -0.70 | 0.06 | 20±14ab | HR | 20±14c | HR |
| *Musa balbiciana* (A&N) | 1 | 5 | 2.3±1.1cd | 2 |  | 0 | 3 | 3 | 1±0.7f | 1 | -1.30 | 0.00 | 40±11cd | R | 20±11f | HR |
| Athiakol-2028 | 2 | 5 | 3.2±0.9d | 3 |  | 0 | 3 | 3 | 2.6±0.8e | 3 | -0.65 | 0.06 | 60±9d | MR | 60±9e | MR |
| Srisailam Collection | 1 | 3 | 2.1±0.6a | 2 |  | 1 | 3 | 3 | 2.5±0.8b | 3 | 0.40 | 0.15 | 40±6a | R | 60±6b | MR |
| Attikol | 3 | 5 | 4±0.7d | 4 |  | 2 | 5 | 5 | 3.9±0.7d | 4 | -0.15 | 0.57 | 80±7d | S | 80±7d | S |
| Jurmony | 3 | 5 | 3.9±0.7a | 4 |  | 2 | 4 | 4 | 3.6±0.6a | 4 | -0.30 | 0.24 | 80±7a | S | 80±7a | S |
| Bhimkol-7 | 1 | 5 | 4.6±1.3a | 5 |  | 5 | 5 | 5 | 5±0a | 5 | 0.40 | 0.16 | 100±13a | HS | 100±13a | HS |
| Athiakol-11 | 1 | 5 | 4.6±1.3cd | 5 |  | 3 | 5 | 5 | 4.4±0.8e | 5 | -0.20 | 0.59 | 100±13cd | HS | 100±13e | HS |
| Bacharia Malbhog | 3 | 5 | 4.8±0.6bc | 5 |  | 5 | 5 | 5 | 5±0d | 5 | 0.20 | 0.16 | 100±6bc | HS | 100±6d | HS |
| Borkal Baista | 3 | 5 | 4.8±0.6bc | 5 |  | 3 | 5 | 5 | 4.5±0.7b | 5 | -0.30 | 0.26 | 100±6bc | HS | 100±6b | HS |
| Elavazhai-555 | 2 | 4 | 3.1±0.7bc | 3 |  | 3 | 5 | 5 | 4.5±0.7b | 5 | 1.40 | 0.00 | 60±7bc | MR | 100±7b | HS |
| Elavazhai-167 | 3 | 5 | 4±0.7ab | 4 |  | 3 | 5 | 5 | 4.3±0.7b | 5 | 0.30 | 0.29 | 80±7ab | S | 100±7b | HS |
| Pagalapahad wild II | 2 | 4 | 3±0.7ab | 3 |  | 0 | 3 | 3 | 1±0.7e | 1 | -2.00 | 0.00 | 60±7ab | MR | 20±7e | HR |
| Jungle Kela II | 0 | 5 | 1.4±1.4c | 1 |  | 0 | 2 | 2 | 0.7±0.6e | 1 | -0.75 | 0.05 | 20±14c | HR | 20±14e | HR |
| H-201 | 0 | 5 | 1.4±1.4c | 1 |  | 0 | 2 | 2 | 0.8±0.6e | 1 | -0.65 | 0.09 | 20±14c | HR | 20±14e | HR |
| Jungle Kela I | 2 | 5 | 3.2±0.9ab | 3 |  | 0 | 2 | 2 | 1.7±0.6d | 2 | -1.55 | 0.00 | 60±9ab | MR | 40±9d | R |
| Khungsang wild | 0 | 4 | 2.7±1.2b | 3 |  | 1 | 3 | 3 | 2.5±0.7c | 3 | -0.25 | 0.46 | 60±12b | MR | 60±12c | MR |
| Phirima wild | 1 | 5 | 3.7±1.1ab | 4 |  | 1 | 4 | 4 | 3.4±0.9b | 4 | -0.30 | 0.42 | 80±11ab | S | 80±11b | S |
| Beejikela | 3 | 5 | 4±0.7a | 4 |  | 5 | 5 | 5 | 5±0a | 5 | 1 | 0.00 | 80±7a | S | 100±7a | HS |
| **AAA Cavendish** |  |  |  |  |  |  |  |  |  |  |  |  |  |  |  |  |
| Shrimanti | 0 | 0 | 0±0d | 0 |  | 0 | 0 | 0 | 0±0e | 0 | 0 | NA | 0±0d | I | 0±0e | I |
| Manjahaji | 0 | 2 | 1±0.5c | 1 |  | 0 | 2 | 2 | 1±0.6d | 1 | 0 | 1.00 | 20±5c | HR | 20±5d | HR |
| Singapur | 1 | 3 | 2±0.7b | 2 |  | 0 | 3 | 3 | 1±0.7d | 1 | -1.00 | 0.00 | 40±7b | R | 20±7d | HR |
| Williams-608 | 1 | 3 | 2±0.5b | 2 |  | 1 | 3 | 3 | 2±0.6c | 2 | 0 | 1.00 | 40±5b | R | 40±5c | R |
| Borjahaji | 2 | 2 | 2±0b | 2 |  | 0 | 4 | 4 | 2.2±0.8c | 2 | 0.15 | 0.57 | 40±0b | R | 40±0c | R |
| GCTCV-119 | 1 | 3 | 2±0.8b | 2 |  | 1 | 3 | 3 | 2±0.6c | 2 | 0 | 1.00 | 40±8b | R | 40±8c | R |
| Highgate | 2 | 4 | 3±0.7a | 3 |  | 1 | 5 | 5 | 3±0.9b | 3 | 0 | 1.00 | 60±7a | MR | 60±7b | MR |
| Mathukar | 2 | 4 | 3±0.7a | 3 |  | 1 | 3 | 3 | 2±0.6c | 2 | -1.00 | 0.00 | 60±7a | MR | 40±7c | R |
| Peddapacha | 1 | 3 | 2±0.7b | 2 |  | 1 | 5 | 5 | 3±1b | 3 | 1 | 0.01 | 40±7b | R | 60±7b | MR |
| GCTCV-215 | 2 | 4 | 3±0.7a | 3 |  | 1 | 5 | 5 | 3±0.9b | 3 | 0 | 1.00 | 60±7a | MR | 60±7b | MR |
| Williams-645 | 1 | 3 | 2±0.5b | 2 |  | 1 | 5 | 5 | 3±0.9b | 3 | 1 | 0.00 | 40±5b | R | 60±5b | MR |
| Jahaji | 1 | 3 | 2±0.8b | 2 |  | 1 | 5 | 5 | 2.9±0.9b | 3 | 0.90 | 0.01 | 40±8b | R | 60±8b | MR |
| Dwarf Cavendish | 2 | 4 | 3±0.7a | 3 |  | 2 | 5 | 5 | 4±0.7a | 4 | 0.95 | 0.00 | 60±7a | MR | 80±7a | S |
| Lacatan | 2 | 4 | 3±0.7a | 3 |  | 1 | 5 | 5 | 4±1a | 4 | 1 | 0.01 | 60±7a | MR | 80±7a | S |
| **AAA Unique** |  |  |  |  |  |  |  |  |  |  |  |  |  |  |  |  |
| Kaveri Sugantham | 1 | 3 | 2±0.7d | 2 |  | 1 | 4 | 4 | 3±0.8b | 3 | 1 | 0.00 | 40±7d | R | 60±7b | MR |
| Leyan | 1 | 3 | 2±0.8d | 2 |  | 2 | 4 | 4 | 3±0.6b | 3 | 1 | 0.00 | 40±8d | R | 60±8b | MR |
| 2390-2 | 2 | 4 | 3±0.7c | 3 |  | 4 | 5 | 5 | 4.9±0.4a | 5 | 1.85 | 0.00 | 60±7c | MR | 100±7a | HS |
| Thellachakarakeli | 2 | 4 | 3±0.8c | 3 |  | 4 | 5 | 5 | 4.9±0.3a | 5 | 1.90 | 0.00 | 60±8c | MR | 100±8a | HS |
| Gros Michel | 3 | 5 | 4±0.7b | 4 |  | 4 | 5 | 5 | 4.9±0.3a | 5 | 0.90 | 0.00 | 80±7b | S | 100±7a | HS |
| Bharat Moni | 5 | 5 | 5±0a | 5 |  | 5 | 5 | 5 | 5±0a | 5 | 0 | 1.00 | 100±0a | HS | 100±0a | HS |
| Red Banana | 0 | 0 | 0±0e | 0 |  | 0 | 0 | 0 | 0±0c | 0 | 0 | NA | 0±0e | I | 0±0c | I |
| **AAB Mysore** |  |  |  |  |  |  |  |  |  |  |  |  |  |  |  |  |
| Chenichampa | 0 | 0 | 0±0e | 0 |  | 0 | 0 | 0 | 0±0e | 0 | 0 | NA | 0±0e | I | 0±0e | I |
| Karpura Chakkrakeli | 0 | 0 | 0±0e | 0 |  | 0 | 0 | 0 | 0±0e | 0 | 0 | NA | 0±0e | I | 0±0e | I |
| Kottavazhai | 0 | 0 | 0±0e | 0 |  | 0 | 0 | 0 | 0±0e | 0 | 0 | NA | 0±0e | I | 0±0e | I |
| Terabun | 0 | 2 | 1±0.7d | 1 |  | 0 | 3 | 3 | 1±0.7d | 1 | 0 | 1.00 | 20±7d | HR | 20±7d | HR |
| Palayankodan | 2 | 4 | 3±0.7b | 3 |  | 1 | 3 | 3 | 2±0.6c | 2 | -1.00 | 0.00 | 60±7b | MR | 40±7c | R |
| Poovan-197 | 0 | 2 | 1±0.7d | 1 |  | 1 | 3 | 3 | 2±0.6c | 2 | 1 | 0.00 | 20±7d | HR | 40±7c | R |
| Poovan-294 | 2 | 4 | 3±0.7b | 3 |  | 1 | 3 | 3 | 2±0.6c | 2 | -1.00 | 0.00 | 60±7b | MR | 40±7c | R |
| Soneri | 0 | 2 | 1±0.5d | 1 |  | 0 | 3 | 3 | 2±0.7c | 2 | 1 | 0.00 | 20±5d | HR | 40±5c | R |
| Mottapoovan | 0 | 2 | 1±0.7d | 1 |  | 1 | 3 | 3 | 2±0.6c | 2 | 1 | 0.00 | 20±7d | HR | 40±7c | R |
| Borchampa | 1 | 3 | 2±0.7c | 2 |  | 2 | 4 | 4 | 3±0.6b | 3 | 1 | 0.00 | 40±7c | R | 60±7b | MR |
| Alpon | 1 | 3 | 2±0.7c | 2 |  | 2 | 4 | 4 | 3±0.6b | 3 | 1 | 0.00 | 40±7c | R | 60±7b | MR |
| Mysorebale | 0 | 2 | 1±0.8d | 1 |  | 2 | 4 | 4 | 3±0.6b | 3 | 2 | 0.00 | 20±8d | HR | 60±8b | MR |
| Pisang Ceylan | 3 | 5 | 4±0.5a | 4 |  | 2 | 5 | 5 | 3±0.7b | 3 | -1.00 | 0.00 | 80±5a | S | 60±5b | MR |
| Garomoina | 2 | 4 | 3±0.5b | 3 |  | 5 | 5 | 5 | 5±0a | 5 | 2 | 0.00 | 60±5b | MR | 100±5a | HS |
| Dasaman | 3 | 5 | 4±0.7a | 4 |  | 5 | 5 | 5 | 5±0a | 5 | 1 | 0.00 | 80±7a | S | 100±7a | HS |
| **AAB Silk** |  |  |  |  |  |  |  |  |  |  |  |  |  |  |  |  |
| Sabri | 1 | 3 | 2.2±0.6d | 2 |  | 0 | 3 | 3 | 2±0.7d | 2 | -0.20 | 0.46 | 40±6d | R | 40±6d | R |
| Bangladesh Malbhog | 2 | 4 | 3±0.7c | 3 |  | 3 | 5 | 5 | 4±0.6c | 4 | 1 | 0.00 | 60±7c | MR | 80±7c | S |
| Malbhog | 2 | 5 | 3.2±0.9c | 3 |  | 5 | 5 | 5 | 5±0a | 5 | 1.80 | 0.00 | 60±9c | MR | 100±9a | HS |
| Dudhsagar-6 | 4 | 5 | 4.9±0.3a | 5 |  | 5 | 5 | 5 | 5±0a | 5 | 0.10 | 0.16 | 100±3a | HS | 100±3a | HS |
| Saapkal | 3 | 5 | 4±0.7b | 4 |  | 5 | 5 | 5 | 5±0a | 5 | 1 | 0.00 | 80±7b | S | 100±7a | HS |
| Digjowa | 3 | 5 | 4±0.7b | 4 |  | 5 | 5 | 5 | 5±0a | 5 | 1 | 0.00 | 80±7b | S | 100±7a | HS |
| Honda | 2 | 5 | 4.1±1.0b | 4 |  | 5 | 5 | 5 | 5±0a | 5 | 0.90 | 0.00 | 80±10b | S | 100±10a | HS |
| Suvandal | 4 | 5 | 4.9±0.3a | 5 |  | 5 | 5 | 5 | 5±0a | 5 | 0.10 | 0.16 | 100±3a | HS | 100±3a | HS |
| Amrithapani-212 | 3 | 5 | 4.1±0.9a | 5 |  | 3 | 5 | 5 | 4.8±0.5ab | 5 | 0.70 | 0.01 | 100±9a | HS | 100±9ab | HS |
| Rasthali | 3 | 5 | 4.8±0.6a | 5 |  | 5 | 5 | 5 | 5±0a | 5 | 0.20 | 0.16 | 100±6a | HS | 100±6a | HS |
| Sakkarchyna | 3 | 5 | 4.2±0.8b | 4 |  | 5 | 5 | 5 | 5±0a | 5 | 0.80 | 0.00 | 80±8b | S | 100±8a | HS |
| Soniyal | 5 | 5 | 5±0a | 5 |  | 5 | 5 | 5 | 5±0a | 5 | 0 | 1.00 | 100±0a | HS | 100±0a | HS |
| Therakanchi | 3 | 5 | 4.8±0.6a | 5 |  | 5 | 5 | 5 | 5±0a | 5 | 0.20 | 0.16 | 100±6a | HS | 100±6a | HS |
| Baidichinia | 2 | 4 | 3.1±0.6c | 3 |  | 5 | 5 | 5 | 5±0a | 5 | 1.90 | 0.00 | 60±6c | MR | 100±6a | HS |
| Ayirankarasthali | 3 | 5 | 4±0.8b | 4 |  | 5 | 5 | 5 | 5±0a | 5 | 1 | 0.00 | 80±8b | S | 100±8a | HS |
| Pisangrajabulu | 3 | 5 | 4±0.8b | 4 |  | 3 | 5 | 5 | 4.7±0.7b | 5 | 0.70 | 0.02 | 80±8b | S | 100±8b | HS |
| Madhuranga | 3 | 5 | 4.1±0.7b | 4 |  | 5 | 5 | 5 | 5±0a | 5 | 0.90 | 0.00 | 80±7b | S | 100±7a | HS |
| Nanjagud rasabale | 5 | 5 | 5±0a | 5 |  | 5 | 5 | 5 | 5±0a | 5 | 0 | 1.00 | 100±0a | HS | 100±0a | HS |
| Thozhuvan | 5 | 5 | 5±0a | 5 |  | 5 | 5 | 5 | 5±0a | 5 | 0 | 1.00 | 100±0a | HS | 100±0a | HS |
| Khozhikodu | 5 | 5 | 5±0a | 5 |  | 5 | 5 | 5 | 5±0a | 5 | 0 | 1.00 | 100±0a | HS | 100±0a | HS |
| Krishnasagar | 5 | 5 | 5±0a | 5 |  | 5 | 5 | 5 | 5±0a | 5 | 0 | 1.00 | 100±0a | HS | 100±0a | HS |
| Ambeli | 5 | 5 | 5±0a | 5 |  | 5 | 5 | 5 | 5±0a | 5 | 0 | 1.00 | 100±0a | HS | 100±0a | HS |
| **AAB Pome** |  |  |  |  |  |  |  |  |  |  |  |  |  |  |  |  |
| Attrusingan | 0 | 1 | 0.1±0.3g | 0 |  | 0 | 0 | 0 | 0±0e | 0 | -0.10 | 0.16 | 0±3g | I | 0±0e | I |
| Ladies Finger-100 | 1 | 3 | 2.1±0.7e | 2 |  | 0 | 3 | 3 | 2±0.7d | 2 | -0.10 | 0.73 | 40±7e | R | 40±7d | R |
| Padathi | 0 | 4 | 2.2±1.2e | 2 |  | 1 | 3 | 3 | 2±0.6d | 2 | -0.20 | 0.56 | 40±12e | R | 40±12d | R |
| Ladan Small | 0 | 3 | 1.9±1.0e | 2 |  | 1 | 3 | 3 | 2±0.6d | 2 | 0.10 | 0.73 | 40±10e | R | 40±10d | R |
| Pacha | 1 | 3 | 2.1±0.7e | 2 |  | 1 | 3 | 3 | 2±0.6d | 2 | -0.10 | 0.71 | 40±7e | R | 40±7d | R |
| H-1 | 0 | 2 | 1.1±0.9f | 2 |  | 1 | 3 | 3 | 2±0.6d | 2 | 0.90 | 0.00 | 40±9f | R | 40±9d | R |
| Chinali-28 | 2 | 4 | 2.9±0.6d | 3 |  | 2 | 4 | 4 | 3±0.6c | 3 | 0.10 | 0.65 | 60±6d | MR | 60±6c | MR |
| Virupakshi | 2 | 5 | 3.8±0.9bc | 4 |  | 2 | 4 | 4 | 3±0.5c | 3 | -0.80 | 0.00 | 80±9bc | S | 60±9c | MR |
| Neyvazhai | 2 | 4 | 3±0.7d | 3 |  | 2 | 4 | 4 | 3±0.6c | 3 | 0 | 1.00 | 60±7d | MR | 60±7c | MR |
| CO-1-164 | 2 | 4 | 3±0.8d | 3 |  | 1 | 4 | 4 | 3±0.6c | 3 | 0 | 1.00 | 60±8d | MR | 60±8c | MR |
| Sirumalai | 2 | 4 | 3.1±0.7cd | 3 |  | 2 | 4 | 4 | 3±0.6c | 3 | -0.10 | 0.71 | 60±7cd | MR | 60±7c | MR |
| Ladanpointed | 1 | 3 | 2.1±0.9e | 3 |  | 2 | 4 | 4 | 3±0.6c | 3 | 0.90 | 0.00 | 60±9e | MR | 60±9c | MR |
| Giant | 2 | 4 | 3±0.8d | 3 |  | 2 | 4 | 4 | 3±0.6c | 3 | 0 | 1.00 | 60±8d | MR | 60±8c | MR |
| Mannan | 2 | 4 | 3.1±0.9cd | 4 |  | 2 | 4 | 4 | 3±0.6c | 3 | -0.10 | 0.71 | 80±9cd | S | 60±9c | MR |
| Co-1-554 | 1 | 4 | 2.1±0.7e | 2 |  | 2 | 4 | 4 | 3±0.6c | 3 | 0.90 | 0.00 | 40±7e | R | 60±7c | MR |
| Marabale | 2 | 5 | 3.2±0.9cd | 3 |  | 2 | 4 | 4 | 3±0.6c | 3 | -0.20 | 0.49 | 60±9cd | MR | 60±9c | MR |
| Malaikali-397 | 2 | 4 | 3±0.8d | 3 |  | 1 | 4 | 4 | 3±0.6c | 3 | 0 | 1.00 | 60±8d | MR | 60±8c | MR |
| H-3 | 0 | 4 | 2.1±1.1e | 2 |  | 2 | 4 | 4 | 3±0.6c | 3 | 0.90 | 0.01 | 40±11e | R | 60±11c | MR |
| Krishnavazhai | 3 | 5 | 4±0.7b | 4 |  | 3 | 5 | 5 | 4±0.5b | 4 | 0 | 1.00 | 80±7b | S | 80±7b | S |
| Chakkarakel-93 | 3 | 5 | 4±0.7b | 4 |  | 2 | 5 | 5 | 3.9±0.6b | 4 | -0.10 | 0.69 | 80±7b | S | 80±7b | S |
| Kallar Ladan | 3 | 5 | 4.8±0.6a | 5 |  | 2 | 5 | 5 | 3.9±0.7b | 4 | -0.95 | 0.00 | 100±6a | HS | 80±6b | S |
| Pachaladan | 2 | 4 | 3±0.7d | 3 |  | 2 | 5 | 5 | 4±0.6b | 4 | 1 | 0.00 | 60±7d | MR | 80±7b | S |
| Ladan | 2 | 5 | 3.2±0.9cd | 3 |  | 3 | 5 | 5 | 4±0.6b | 4 | 0.80 | 0.01 | 60±9cd | MR | 80±9b | S |
| Malaikali-113 | 3 | 5 | 4±0.5b | 4 |  | 2 | 5 | 5 | 4±0.6b | 4 | 0 | 1.00 | 80±5b | S | 80±5b | S |
| Thenkali | 3 | 5 | 3.9±0.7b | 4 |  | 3 | 5 | 5 | 4±0.6b | 4 | 0.10 | 0.68 | 80±7b | S | 80±7b | S |
| Ennabenian-489 | 2 | 4 | 3±0.7d | 3 |  | 2 | 5 | 5 | 3.7±0.8b | 4 | 0.70 | 0.02 | 60±7d | MR | 80±7b | S |
| Ladies Finger-280 | 2 | 4 | 3±0.7d | 3 |  | 3 | 5 | 5 | 4±0.6b | 4 | 1 | 0.00 | 60±7d | MR | 80±7b | S |
| Peykadali | 4 | 5 | 4.9±0.3a | 5 |  | 3 | 5 | 5 | 4±0.6b | 4 | -0.90 | 0.00 | 100±3a | HS | 80±3b | S |
| Chakarakel-376 | 3 | 5 | 4±0.5b | 4 |  | 3 | 5 | 5 | 4±0.6b | 4 | 0 | 1.00 | 80±5b | S | 80±5b | S |
| Hoobale | 2 | 5 | 3.2±0.9cd | 3 |  | 3 | 5 | 5 | 4±0.6b | 4 | 0.80 | 0.01 | 60±9cd | MR | 80±9b | S |
| Padathi-537 | 2 | 5 | 3.2±0.9cd | 3 |  | 2 | 5 | 5 | 3.7±0.8b | 4 | 0.45 | 0.18 | 60±9cd | MR | 80±9b | S |
| Figue Pomme Geante | 2 | 4 | 3.1±0.7cd | 3 |  | 3 | 5 | 5 | 4.1±0.6b | 4 | 0.95 | 0.00 | 60±7cd | MR | 80±7b | S |
| Numaran | 3 | 5 | 4±0.5b | 4 |  | 3 | 5 | 5 | 4±0.6b | 4 | 0 | 1.00 | 80±5b | S | 80±5b | S |
| Vannan | 3 | 5 | 4.1±0.6b | 4 |  | 5 | 5 | 5 | 5±0a | 5 | 0.90 | 0.00 | 80±6b | S | 100±6a | HS |
| Kaali | 5 | 5 | 5±0a | 5 |  | 4 | 5 | 5 | 4.9±0.4a | 5 | -0.15 | 0.21 | 100±0a | HS | 100±0a | HS |
| Malaikali-125 | 5 | 5 | 5±0a | 5 |  | 4 | 5 | 5 | 4.9±0.4a | 5 | -0.15 | 0.21 | 100±0a | HS | 100±0a | HS |
| Malaivazhai | 3 | 5 | 4.1±0.7b | 4 |  | 4 | 5 | 5 | 4.9±0.4a | 5 | 0.75 | 0.00 | 80±7b | S | 100±7a | HS |
| **AAB Unique** |  |  |  |  |  |  |  |  |  |  |  |  |  |  |  |  |
| Jwaribale | 5 | 5 | 5±0a | 5 |  | 2 | 4 | 4 | 3±0.5c | 3 | -2.00 | 0.00 | 100±0a | HS | 60±0c | MR |
| Kullan | 3 | 5 | 4.8±0.6a | 5 |  | 3 | 5 | 5 | 4.8±0.6a | 5 | -0.05 | 0.82 | 100±6a | HS | 100±6a | HS |
| Cherapadathi | 5 | 5 | 5±0a | 5 |  | 3 | 5 | 5 | 4±0.6b | 4 | -1.00 | 0.00 | 100±0a | HS | 80±0b | S |
| Nendrapadathi | 1 | 4 | 2.8±0.8b | 3 |  | 3 | 5 | 5 | 4±0.6b | 4 | 1.20 | 0.00 | 60±8b | MR | 80±8b | S |
| Nendrakali | 5 | 5 | 5±0a | 5 |  | 3 | 5 | 5 | 4.7±0.7a | 5 | -0.30 | 0.16 | 100±0a | HS | 100±0a | HS |
| Popoulou | 0 | 5 | 1.4±1.3c | 1 |  | 0 | 2 | 2 | 1±0.5e | 1 | -0.40 | 0.24 | 20±13c | HR | 20±13e | HR |
| Kalibow | 5 | 5 | 5±0a | 5 |  | 1 | 3 | 3 | 2±0.6d | 2 | -3.00 | 0.00 | 100±0a | HS | 40±0d | R |
| Pisang Rajah | 3 | 5 | 4.8±0.6a | 5 |  | 2 | 4 | 4 | 3±0.5c | 3 | -1.80 | 0.00 | 100±6a | HS | 60±6c | MR |
| Dudhsagar-374 | 5 | 5 | 5±0a | 5 |  | 2 | 4 | 4 | 3±0.6c | 3 | -2.00 | 0.00 | 100±0a | HS | 60±0c | MR |
| Thiruvanthapuram | 2 | 5 | 3.2±0.8b | 3 |  | 3 | 5 | 5 | 4±0.6b | 4 | 0.80 | 0.01 | 60±8b | MR | 80±8b | S |
| Pisang Seribu | 1 | 5 | 4.6±1.3a | 5 |  | 4 | 5 | 5 | 4.8±0.4a | 5 | 0.20 | 0.52 | 100±13a | HS | 100±13a | HS |
| Chinali-483 | 0 | 5 | 4.5±1.6a | 5 |  | 5 | 5 | 5 | 5±0a | 5 | 0.50 | 0.16 | 100±16a | HS | 100±16a | HS |
| **AAB Plantain** |  |  |  |  |  |  |  |  |  |  |  |  |  |  |  |  |
| Nijokome | 0 | 2 | 0.9±0.6a | 1 |  | 0 | 2 | 2 | 1±0.6a | 1 | 0.10 | 0.65 | 20±6a | HR | 20±6a | HR |
| Chengalikodan | 0 | 0 | 0±0b | 0 |  | 0 | 0 | 0 | 0±0b | 0 | 0 | NA | 0±0b | I | 0±0b | I |
| Nendran-296 | 0 | 3 | 0.3±0.9b | 0 |  | 0 | 0 | 0 | 0±0b | 0 | -0.30 | 0.16 | 0±0b | I | 0±0b | I |
| Nendren-615 | 0 | 4 | 0.4±1.3b | 0 |  | 0 | 0 | 0 | 0±0b | 0 | -0.40 | 0.16 | 0±13b | I | 0±0b | I |
| Nedu Nendran | 0 | 4 | 0.4±1.3b | 0 |  | 0 | 0 | 0 | 0±0b | 0 | -0.40 | 0.16 | 0±13b | I | 0±0b | I |
| **ABB Pisang Awak** |  |  |  |  |  |  |  |  |  |  |  |  |  |  |  |  |
| Desshikadali | 5 | 5 | 5±0a | 5 |  | 2 | 5 | 5 | 3.8±0.7c | 4 | -1.25 | 0.00 | 100±0a | HS | 80±0c | S |
| Agnimalbhog | 5 | 5 | 5±0a | 5 |  | 5 | 5 | 5 | 5±0a | 5 | 0 | 1.00 | 100±0a | HS | 100±0a | HS |
| Kanthali | 3 | 5 | 4.1±0.7bc | 4 |  | 3 | 5 | 5 | 4.7±0.6b | 5 | 0.55 | 0.03 | 80±7bc | S | 100±7b | HS |
| Chinia-87 | 5 | 5 | 5±0a | 5 |  | 5 | 5 | 5 | 5±0a | 5 | 0 | 1.00 | 100±0a | HS | 100±0a | HS |
| BatisaPiro | 5 | 5 | 5±0a | 5 |  | 5 | 5 | 5 | 5±0a | 5 | 0 | 1.00 | 100±0a | HS | 100±0a | HS |
| Ennabenian-103 | 5 | 5 | 5±0a | 5 |  | 3 | 5 | 5 | 4.6±0.7b | 5 | -0.40 | 0.08 | 100±0a | HS | 100±0b | HS |
| Shailkela | 5 | 5 | 5±0a | 5 |  | 3 | 5 | 5 | 4.5±0.8b | 5 | -0.50 | 0.05 | 100±0a | HS | 100±0b | HS |
| Nepalichinia-117 | 4 | 5 | 4.9±0.3ab | 5 |  | 3 | 5 | 5 | 4.6±0.7b | 5 | -0.30 | 0.20 | 100±3ab | HS | 100±3b | HS |
| Bhurkel | 5 | 5 | 5±0a | 5 |  | 5 | 5 | 5 | 5±0a | 5 | 0 | 1.00 | 100±0a | HS | 100±0a | HS |
| Karpuravalli-173 | 5 | 5 | 5±0a | 5 |  | 3 | 5 | 5 | 4.5±0.7b | 5 | -0.50 | 0.03 | 100±0a | HS | 100±0b | HS |
| Vellapalayankodan | 5 | 5 | 5±0a | 5 |  | 5 | 5 | 5 | 5±0a | 5 | 0 | 1.00 | 100±0a | HS | 100±0a | HS |
| Jamulapalem Collection | 5 | 5 | 5±0a | 5 |  | 3 | 5 | 5 | 4.7±0.6b | 5 | -0.35 | 0.07 | 100±0a | HS | 100±0b | HS |
| Karpuravalli-291 | 5 | 5 | 5±0a | 5 |  | 5 | 5 | 5 | 5±0a | 5 | 0 | 1.00 | 100±0a | HS | 100±0a | HS |
| Gauria | 4 | 5 | 4.9±0.3ab | 5 |  | 5 | 5 | 5 | 5±0a | 5 | 0.10 | 0.16 | 100±3ab | HS | 100±3a | HS |
| Chinia-347 | 3 | 5 | 4±0.7c | 4 |  | 3 | 5 | 5 | 4.6±0.7b | 5 | 0.60 | 0.03 | 80±7c | S | 100±7b | HS |
| Vananthpurani | 5 | 5 | 5±0a | 5 |  | 5 | 5 | 5 | 5±0a | 5 | 0 | 1.00 | 100±0a | HS | 100±0a | HS |
| DakshinSagar | 5 | 5 | 5±0a | 5 |  | 3 | 5 | 5 | 4.7±0.7b | 5 | -0.35 | 0.11 | 100±0a | HS | 100±0b | HS |
| Ladisan | 5 | 5 | 5±0a | 5 |  | 5 | 5 | 5 | 5±0a | 5 | 0 | 1.00 | 100±0a | HS | 100±0a | HS |
| Poombidiyan | 5 | 5 | 5±0a | 5 |  | 3 | 5 | 5 | 4.7±0.6ab | 5 | -0.30 | 0.11 | 100±0a | HS | 100±0ab | HS |
| Bankela | 5 | 5 | 5±0a | 5 |  | 5 | 5 | 5 | 5±0a | 5 | 0 | 1.00 | 100±0a | HS | 100±0a | HS |
| Karpuravalli-494 | 3 | 5 | 4.1±0.7bc | 4 |  | 3 | 5 | 5 | 4.7±0.6ab | 5 | 0.60 | 0.02 | 80±7bc | S | 100±7ab | HS |
| Boothibale | 3 | 5 | 4.1±0.7bc | 4 |  | 5 | 5 | 5 | 5±0a | 5 | 0.90 | 0.00 | 80±7bc | S | 100±7a | HS |
| Enikomban | 1 | 5 | 4.6±1.3abc | 5 |  | 5 | 5 | 5 | 5±0a | 5 | 0.40 | 0.16 | 100±13abc | HS | 100±13a | HS |
| Amrithapani-734 | 0 | 5 | 4.5±1.6abc | 5 |  | 5 | 5 | 5 | 5±0a | 5 | 0.50 | 0.16 | 100±16abc | HS | 100±16a | HS |
| Ankur-I | 1 | 5 | 4.6±1.3abc | 5 |  | 5 | 5 | 5 | 5±0a | 5 | 0.40 | 0.16 | 100±13abc | HS | 100±13a | HS |
| Dinamalakol | 0 | 5 | 4.5±1.6abc | 5 |  | 5 | 5 | 5 | 5±0a | 5 | 0.50 | 0.16 | 100±16abc | HS | 100±16a | HS |
| GP-24 | 0 | 5 | 4.5±1.6abc | 5 |  | 5 | 5 | 5 | 5±0a | 5 | 0.50 | 0.16 | 100±16abc | HS | 100±16a | HS |
| NIC-2046 | 0 | 5 | 4.5±1.6abc | 5 |  | 3 | 5 | 5 | 4.7±0.6ab | 5 | 0.20 | 0.61 | 100±16abc | HS | 100±16ab | HS |
| Octoman | 0 | 5 | 4.5±1.6abc | 5 |  | 5 | 5 | 5 | 5±0a | 5 | 0.50 | 0.16 | 100±16abc | HS | 100±16a | HS |
| **ABB Monthan** |  |  |  |  |  |  |  |  |  |  |  |  |  |  |  |  |
| Chirapunji-525 | 3 | 5 | 4.1±0.7b | 4 |  | 2 | 5 | 5 | 3.7±0.9c | 4 | 0 | 1.00 | 100±0a | HS | 100±0a | HS |
| Kachkel | 5 | 5 | 5±0a | 5 |  | 5 | 5 | 5 | 5±0a | 5 | -0.55 | 0.02 | 100±3a | HS | 80±3b | S |
| Bankel | 4 | 5 | 4.9±0.3a | 5 |  | 3 | 5 | 5 | 4.4±0.7b | 4 | 0 | 1.00 | 100±0a | HS | 100±0a | HS |
| Pidimonthan | 5 | 5 | 5±0a | 5 |  | 5 | 5 | 5 | 5±0a | 5 | 0.10 | 0.16 | 100±3a | HS | 100±3a | HS |
| Karimbontha-121 | 4 | 5 | 4.9±0.3a | 5 |  | 5 | 5 | 5 | 5±0a | 5 | 0 | 1.00 | 100±0a | HS | 100±0a | HS |
| Karibale-129 | 5 | 5 | 5±0a | 5 |  | 5 | 5 | 5 | 5±0a | 5 | 0 | 1.00 | 100±0a | HS | 100±0a | HS |
| Lamby | 5 | 5 | 5±0a | 5 |  | 5 | 5 | 5 | 5±0a | 5 | 0 | 1.00 | 100±0a | HS | 100±0a | HS |
| Kallumonthan | 5 | 5 | 5±0a | 5 |  | 5 | 5 | 5 | 5±0a | 5 | 0 | 1.00 | 80±7b | S | 80±7c | S |
| Bathesa ash | 3 | 5 | 4±0.7b | 4 |  | 3 | 5 | 5 | 4±0.7c | 4 | 0 | 1.00 | 100±0a | HS | 100±0a | HS |
| Monthan | 5 | 5 | 5±0a | 5 |  | 5 | 5 | 5 | 5±0a | 5 | 0.10 | 0.75 | 80±7b | S | 100±7b | HS |
| Peykunnan-338 | 3 | 5 | 4.1±0.7b | 4 |  | 3 | 5 | 5 | 4.2±0.8b | 5 | 0 | 1.00 | 100±0a | HS | 100±0a | HS |
| Mathokgrang | 5 | 5 | 5±0a | 5 |  | 5 | 5 | 5 | 5±0a | 5 | 0.10 | 0.16 | 100±3a | HS | 100±3a | HS |
| Karibale-518 | 4 | 5 | 4.9±0.3a | 5 |  | 5 | 5 | 5 | 5±0a | 5 | 0 | 1.00 | 100±0a | HS | 100±0a | HS |
| Kosthabontha | 5 | 5 | 5±0a | 5 |  | 5 | 5 | 5 | 5±0a | 5 | 0.90 | 0.00 | 80±7b | S | 100±7a | HS |
| Peykunnan-538 | 3 | 5 | 4.1±0.7b | 4 |  | 5 | 5 | 5 | 5±0a | 5 | 0 | 1.00 | 100±0a | HS | 100±0a | HS |
| Chetty | 5 | 5 | 5±0a | 5 |  | 5 | 5 | 5 | 5±0a | 5 | 0 | 1.00 | 100±0a | HS | 100±0a | HS |
| Goukar | 5 | 5 | 5±0a | 5 |  | 5 | 5 | 5 | 5±0a | 5 | 0.15 | 0.62 | 80±7ab | S | 100±7b | HS |
| Sambrani Monthan | 5 | 5 | 5±0a | 5 |  | 5 | 5 | 5 | 5±0a | 5 | 0 | 1.00 | 100±0a | HS | 100±0a | HS |
| Birbutia | 3 | 5 | 4.1±0.7ab | 4 |  | 3 | 5 | 5 | 4.3±0.8b | 5 | 0.10 | 0.16 | 100±3a | HS | 100±3a | HS |
| Batisa local | 5 | 5 | 5±0a | 5 |  | 5 | 5 | 5 | 5±0a | 5 | 0 | 1.00 | 100±0a | HS | 100±0a | HS |
| Pacha Bontha Batheesa | 4 | 5 | 4.9±0.3a | 5 |  | 5 | 5 | 5 | 5±0a | 5 | 0 | 1.00 | 100±0a | HS | 100±0a | HS |
| Booditha Bontha Batheesa | 5 | 5 | 5±0a | 5 |  | 5 | 5 | 5 | 5±0a | 5 | -0.45 | 0.17 | 80±7b | S | 80±7c | S |
| Ashy Batheesa | 5 | 5 | 5±0a | 5 |  | 5 | 5 | 5 | 5±0a | 5 | 0 | 1.00 | 100±0a | HS | 100±0a | HS |
| Nutepong | 5 | 5 | 5±0a | 5 |  | 3 | 5 | 5 | 4.5±0.7b | 5 | -0.50 | 0.03 | 100±0a | HS | 100±0b | HS |
| Kait Khullung | 5 | 5 | 5±0a | 5 |  | 5 | 5 | 5 | 5±0a | 5 | 0 | 1.00 | 100±0a | HS | 100±0a | HS |
| Kait Shejing | 5 | 5 | 5±0a | 5 |  | 5 | 5 | 5 | 5±0a | 5 | 0 | 1.00 | 100±0a | HS | 100±0a | HS |
| Singhalaji | 3 | 5 | 4.1±0.7b | 4 |  | 5 | 5 | 5 | 5±0a | 5 | 0.90 | 0.00 | 80±7b | S | 100±7a | HS |
| Chirapunji-344 | 5 | 5 | 5±0a | 5 |  | 5 | 5 | 5 | 5±0a | 5 | 0 | 1.00 | 100±0a | HS | 100±0a | HS |
| Bainsa | 5 | 5 | 5±0a | 5 |  | 5 | 5 | 5 | 5±0a | 5 | 0 | 1.00 | 100±0a | HS | 100±0a | HS |
| Ney vannan | 5 | 5 | 5±0a | 5 |  | 5 | 5 | 5 | 5±0a | 5 | 0 | 1.00 | 100±0a | HS | 100±0a | HS |
| Vennut Mannan-251 | 5 | 5 | 5±0a | 5 |  | 5 | 5 | 5 | 5±0a | 5 | 0 | 1.00 | 100±0a | HS | 100±0a | HS |
| Vennut Mannan-26 | 5 | 5 | 5±0a | 5 |  | 5 | 5 | 5 | 5±0a | 5 | 0 | 1.00 | 100±0a | HS | 100±0a | HS |
| Cuba | 4 | 5 | 4.9±0.3a | 5 |  | 5 | 5 | 5 | 5±0a | 5 | 0.10 | 0.16 | 100±3a | HS | 100±3a | HS |
| Karimbontha-413 | 5 | 5 | 5±0a | 5 |  | 5 | 5 | 5 | 5±0a | 5 | 0 | 1.00 | 100±0a | HS | 100±0a | HS |
| Manjavazhai | 5 | 5 | 5±0a | 5 |  | 5 | 5 | 5 | 5±0a | 5 | 0 | 1.00 | 100±0a | HS | 100±0a | HS |
| Bluggoe | 5 | 5 | 5±0a | 5 |  | 5 | 5 | 5 | 5±0a | 5 | 0 | 1.00 | 100±0a | HS | 100±0a | HS |
| Bersain | 5 | 5 | 5±0a | 5 |  | 5 | 5 | 5 | 5±0a | 5 | 0 | 1.00 | 100±0a | HS | 100±0a | HS |
| Beula | 5 | 5 | 5±0a | 5 |  | 5 | 5 | 5 | 5±0a | 5 | 0 | 1.00 | 100±0a | HS | 100±0a | HS |
| Kothia | 1 | 5 | 3.7±1.1b | 4 |  | 5 | 5 | 5 | 5±0a | 5 | 1.30 | 0.00 | 80±11b | S | 100±11a | HS |
| Nepali Chinia-97 | 5 | 5 | 5±0a | 5 |  | 3 | 5 | 5 | 4.4±0.7b | 5 | -0.65 | 0.01 | 100±0a | HS | 100±0b | HS |
| Kapur | 5 | 5 | 5±0a | 5 |  | 5 | 5 | 5 | 5±0a | 5 | 0 | 1.00 | 100±0a | HS | 100±0a | HS |
| Bungan | 3 | 5 | 4.8±0.6a | 5 |  | 5 | 5 | 5 | 5±0a | 5 | 0.20 | 0.16 | 100±6a | HS | 100±6a | HS |
| Bangrier | 0 | 5 | 4.5±1.6ab | 5 |  | 5 | 5 | 5 | 5±0a | 5 | 0.50 | 0.16 | 100±16ab | HS | 100±16a | HS |
| Chakkiya | 0 | 5 | 4.5±1.6ab | 5 |  | 5 | 5 | 5 | 5±0a | 5 | 0.50 | 0.16 | 100±16ab | HS | 100±16a | HS |
| **ABB Unique** |  |  |  |  |  |  |  |  |  |  |  |  |  |  |  |  |
| Karthobium | 0 | 2 | 1±0.7c | 1 |  | 0 | 2 | 2 | 1.5±0.7c | 2 | 0.50 | 0.07 | 20±7c | HR | 40±7c | R |
| Kaitkhullung | 2 | 5 | 4.7±0.9a | 5 |  | 5 | 5 | 5 | 5±0a | 5 | 0.30 | 0.16 | 100±9a | HS | 100±9a | HS |
| Monthan II | 3 | 5 | 4.8±0.6a | 5 |  | 5 | 5 | 5 | 5±0a | 5 | 0.20 | 0.16 | 100±6a | HS | 100±6a | HS |
| Ginde | 2 | 4 | 2.9±0.7b | 3 |  | 2 | 4 | 4 | 3.5±0.8b | 4 | 0.55 | 0.07 | 60±7b | MR | 80±7b | S |
| **Tetraploid** |  |  |  |  |  |  |  |  |  |  |  |  |  |  |  |  |
| Sawai | 2 | 5 | 3.2±0.9c | 3 |  | 2 | 4 | 4 | 3.5±0.7c | 4 | 0.30 | 0.32 | 60±9c | MR | 80±9c | S |
| Hybrid Sawai | 5 | 5 | 5±0a | 5 |  | 5 | 5 | 5 | 5±0a | 5 | 0 | 1.00 | 100±0a | HS | 100±0a | HS |
| Klue teprod | 4 | 5 | 4.9±0.3a | 5 |  | 5 | 5 | 5 | 5±0a | 5 | 0.10 | 0.16 | 100±3a | HS | 100±3a | HS |
| Klue teparod | 5 | 5 | 5±0a | 5 |  | 5 | 5 | 5 | 5±0a | 5 | 0 | 1.00 | 100±0a | HS | 100±0a | HS |
| TMB× 5295-1 | 0 | 4 | 0.4±1.3e | 0 |  | 0 | 0 | 0 | 0±0e | 0 | -0.40 | 0.16 | 0±13e | I | 0±0e | I |
| FHIA-01 | 0 | 4 | 1.3±1.2d | 1 |  | 0 | 2 | 2 | 1.5±0.7d | 2 | 0.20 | 0.56 | 20±12d | HR | 40±12d | R |
| Foconah | 2 | 5 | 4±0.8b | 4 |  | 3 | 5 | 5 | 4.6±0.7b | 5 | 0.55 | 0.06 | 80±8b | S | 100±8b | HS |

Means with the same letter in the column of respective genomic group are not significantly different (P>0.05) in pairwise mean comparison of crosses by Duncan's multiple range test (DMRT) test. DI= Disease Index, DR= Disease Reaction categories.
